# Computed tomography derived analytic morphomics as predictors of clinical outcomes in trauma: a systematic narrative review

**DOI:** 10.1007/s10140-026-02441-x

**Published:** 2026-02-07

**Authors:** Ludolf G.A. De Kock, Ronan J. Lee, David O. Adebayo, Patrick D. Mclaughin, Eanna MacSuibhne, Michael M. Maher, David J. Ryan

**Affiliations:** 1 Department of Radiology, Cork University Hospital, Cork, Ireland; 2 Department of Radiology, School of Medicine, University College Cork, Cork, Ireland; 3 Department of Radiography, University College Cork, Cork, Ireland; 4 Department of Emergency Medicine, Cork University Hospital, Cork, Ireland

**Keywords:** Trauma, Body composition, Computed tomography, Sarcopenia, Bone mineral density, Patient outcome assessment

## Abstract

Medical imaging plays a central role in the management of trauma patients. Analytic morphomics (AM) through enabling measurement of specific biological markers of body composition from medical images is emerging as a potential tool to predict patient outcomes across multiple medical and surgical disciplines. We sought to provide a comprehensive review of the utility of AM in predicting outcomes in trauma patients. A systematic review with a narrative synthesis was conducted following the Preferred Reporting Items for Systematic Review and Meta-analyses (PRISMA) guidelines and checklists. PubMed, Embase, Scopus, The Cochrane Library as well as Web of Science and Trip database were searched for studies that assessed the relationship between computed tomography-based AM parameters and clinical outcomes in patients from a trauma cohort. Multiple AM domains, including lumbar muscle quantity and quality, adiposity, craniofacial measurements, and opportunistic bone mineral density (BMD), were consistently associated with adverse outcomes including mortality, length of stay, complications, and functional recovery. CT-derived AM metrics provide valuable prognostic information in trauma populations, extending beyond conventional measures such as chronological age and injury severity scores.

**Registration**: PROSPERO Registration ID: CRD420251112652

**Graphical abstract:**

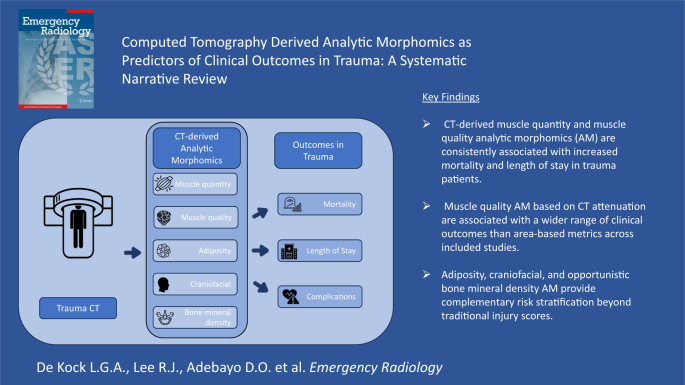

**Supplementary Information:**

The online version contains supplementary material available at 10.1007/s10140-026-02441-x.

## Introduction

Trauma remains a leading cause of morbidity and mortality globally, with the World Health Organisation reporting ~ 4.4 million deaths annually [1]. Improvements in multidisciplinary trauma care have reduced mortality from 19% in the 1990 s to ~ 12% in 2020 across Europe [2]. Medical imaging is central to trauma management, enabling rapid diagnosis of life-threatening injuries. Whole-body computed tomography (WBCT) is particularly valuable in severely injured patients not requiring immediate surgery, improving detection of serious injuries and reducing mortality compared with selective imaging [3, 4]. This has contributed to increasing CT use in emergency departments, especially among older patients [5, 6].

The proportion of people ≥ 65 years in the European Union has risen from 18.7% in 2014 to 21.6% in 2024 [7]. Consequently, more older adults present with major trauma. Advancing age is a strong predictor of intensive care unit (ICU) admission, prolonged hospital length of stay (LOS), and worse outcomes, with patients ≥ 65 at highest risk [8]. Sarcopenia—progressive skeletal muscle loss linked to ageing and chronic illness—has been identified as a contributing factor [9–11].

Analytic morphomics (AM), a CT-based body composition assessment technique derived from existing imaging, is increasingly applied across trauma, surgery, and oncology populations to predict patient outcome [12]. Measurements of psoas muscle area and density on CT slices provide objective markers of sarcopenia [13, 14], while adiposity parameters such as subcutaneous and visceral fat also predict surgical outcomes [15, 16].

Although extensively studied in surgical research, few studies have examined its role in trauma. Surgical cohorts often differ from trauma populations in physiological instability, injury severity, and acute metabolic responses, limiting direct translation of findings [17]. In trauma care, WBCT is routinely obtained as part of initial assessment, allowing AM-derived metrics to be used opportunistically—without additional imaging—and incorporated into clinical decision-making, underscoring its practicality in Emergency Radiology.

Although demographic examples presented above focus mainly on European populations, rising demand for emergency care services has also been widely reported across OECD countries outside Europe, reflecting increasing global demand on trauma systems [18]. While older adults (> 65 years) constitute a rapidly growing trauma demographic, this review aims to comprehensively assess AM in all adult trauma populations (> 16 years) globally, and its ability to predict LOS, complications, ICU admission, and mortality.

## Methods

A systematic review with narrative synthesis was performed according to PRISMA guidelines [19]. We searched PubMed, Embase, Scopus, Cochrane Library, Web of Science, and Trip. No date limits were applied, and the final search was completed in July 2025. Eligible studies included observational designs (prospective and retrospective cohort studies and case–control studies) assessing relationships between CT-derived AM parameters and trauma outcomes. Two independent reviewers conducted the search. Search terms included *analytic morphomics*, *CT analytic morphomics*, *trauma*, *injury*, and *outcomes*, combined with Boolean operators and relevant Medical Subject Headings (MeSH) including *Body Composition and Patient Outcome Assessment*. The review protocol was registered on PROSPERO (*CRD420251112652*), with the full search strategy available at: https://www.crd.york.ac.uk/PROSPERO/view/CRD420251112652*.* Reference lists of eligible studies were also screened.

Eligible populations included adult patients (> 16 years) with trauma in whom AM parameters (e.g., psoas muscle index [PMI], psoas cross-sectional area [PCSA], visceral fat area [VFA]) were assessed for outcomes such as mortality, ICU or hospital length of stay (LOS), and complications. AM parameters derived from both whole-body CT (WBCT) and regional CT were included, as clinical practice often involves an overlap between these modalities and discrepancies in how trauma imaging is performed across varying trauma centres. Although the introduction highlights older adults as a key demographic, the review intentionally included all adults to ensure a complete evaluation of AM parameters across the full adult trauma cohort. Only English-language studies were included. Exclusion criteria were paediatric populations, studies lacking outcomes linked to AM, non-trauma cohorts, case reports, editorials, letters, or dissertations.

Title and abstract screening and full-text review were independently performed by two blinded authors, with disagreements resolved by a senior investigator. Screening was completed in Rayyan.ai (Qatar Computing Research Institute, Doha, Qatar), and data (authors, population, demographics, cohort size, outcomes) were extracted into Microsoft Excel (Microsoft Corp., Redmond, WA, USA). Study quality was independently calculated by two reviewers using the Newcastle-Ottawa Scale (NOS), evaluating studies across the selection, comparability, and outcome domains (Supplementary Table 1). Discrepancies in scoring were resolved by consensus [20]. Minor language editing was performed using ChatGPT (OpenAI, San Francisco, CA, USA), with all content verified by the authors.

## Results

**Fig. 1:**
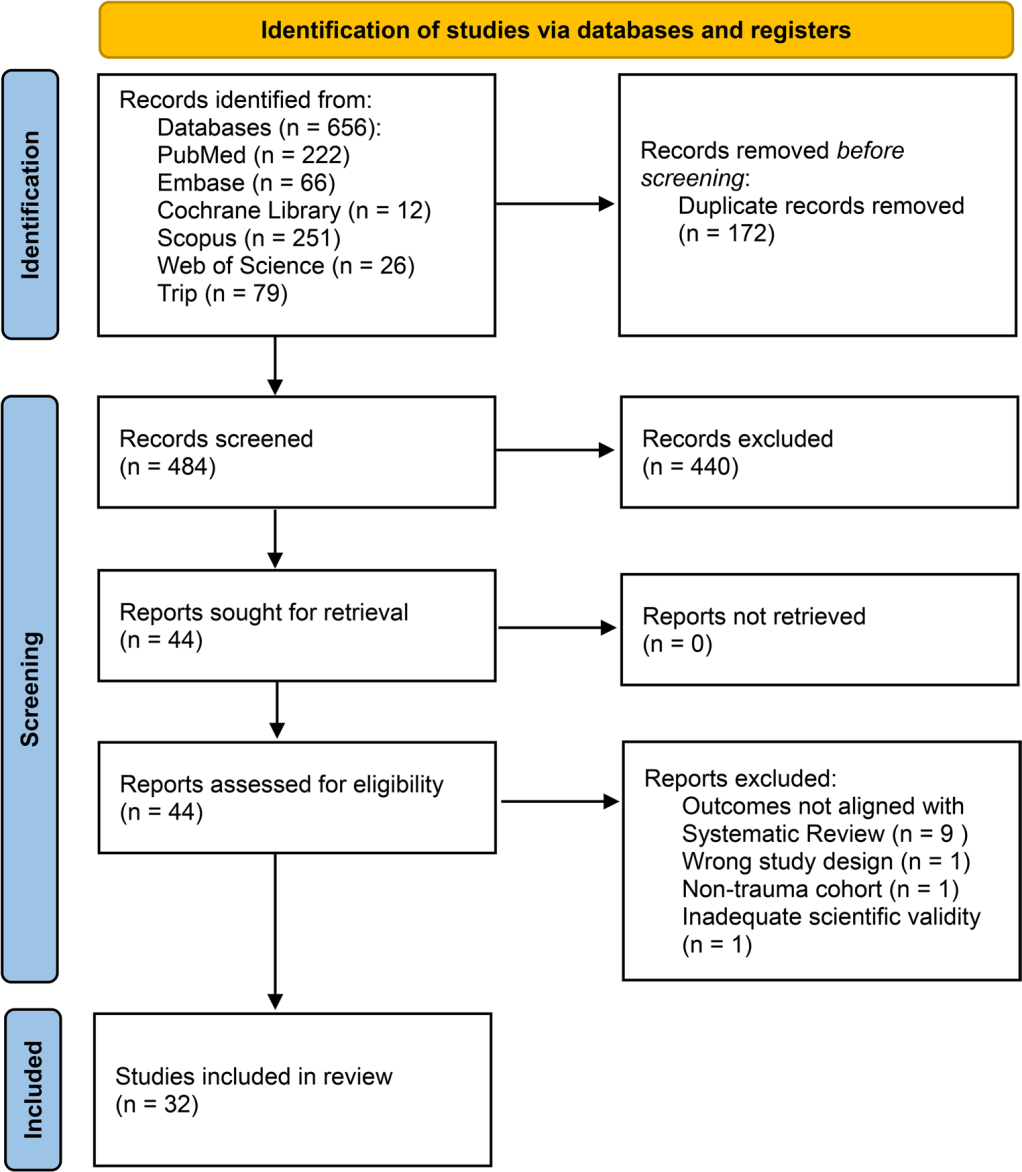
PRISMA Flow-diagram of study selection

A systematic search of six databases (PubMed, Embase, Cochrane Library, Scopus, Web of Science, and Trip) identified 656 records. After removing 172 duplicates, 484 records were screened by title and abstract, with 440 excluded. Forty-four full texts were assessed for eligibility; 12 were excluded (nine for unrelated outcomes, one for wrong study design, one for a non-trauma cohort, and one with inadequate scientific validity). Ultimately, 32 studies met inclusion criteria and were included in the review (Fig. 1).

The included studies were primarily organised according to AM measurement domain, which formed the dominant framework for synthesis in this review. Five AM domains were identified: lumbar muscle quantity, lumbar muscle quality, adiposity, craniofacial and opportunistic bone mineral density. Lumbar muscle quantity AM were most frequent, with 23 studies (71.9%) assessing psoas or skeletal muscle. Lumbar muscle quality AM using CT attenuation were utilised in five studies (15.6%), typically psoas or skeletal muscle density. Adiposity AM were reported in seven studies (21.9%), mainly visceral fat area and subcutaneous fat and combined ratios. Craniofacial AM from head CTs (masseter or temporalis muscles) featured in five studies (15.6%), and 4 studies (12.5%) assessed opportunistic bone mineral density AM. Several studies evaluated more than one AM domain.

Within each AM domain, reported clinical outcomes were assessed across three clinical categories: mortality, ICU and hospital length of stay, and complications and other adverse outcomes, including functional status, discharge destination, and complication rates.

## Lumbar muscle quantity analytic morphomics

CT-derived lumbar muscle quantity metrics were the most frequently applied analytic morphomic measures across included studies. Despite substantial heterogeneity in populations, trauma mechanisms, and measurement approaches, findings could be meaningfully synthesised across three outcome domains: mortality, length of stay (LOS), and adverse outcomes or complications. Table 1 summarises the characteristics of studies assessing lumbar muscle quantity analytic morphomics, including cohort characteristics, measurement level, analytic approach, and associated outcomes.

**Table 1:** Lumbar muscle quantity analytic morphomics

Study Name	Author	Date	Cohort (*n*)	Population	Level Measured	Software	Analytic Morphomic Variables	Conclusion
*Acute post-traumatic muscle atrophy on CT scan predicts prolonged mechanical ventilation and a worse outcome in severe trauma patients*	Tazerout et al.	2022	114	All Trauma ICU	L3	Local Picture Archiving and Communication System (PACS) workstation – manual outlining	Delta psoas muscle index (ΔPMI) % decline, APTMA (acute post-traumatic muscle atrophy)	A greater decrease in APTMA (acute post-traumatic muscle atrophy) was strongly associated with longer mechanical ventilation, prolonged ICU/hospital stay, and higher complication rates in severe trauma patients.
*Based on CT scans at the 12th thoracic spine level*,* assessing the impact of skeletal muscle and adipose tissue index on one-year postoperative mortality in elderly hip fracture patients: a propensity score-matched multicenter retrospective study*	Li et al.	2025	334	Hip fracture post-surgery	T12	ImageJ (National Institute of Health Image program v1.52c – manual/semi-automated segmentation)	Skeletal muscle index (SMI)	SMI independently predicted higher one-year postoperative mortality in elderly hip fracture patients.
*Body composition parameters in initial CT imaging of mechanically ventilated trauma patients: Single-centre observational study*	Meyer et al.	2024	472	Ventilated trauma ICU	L3	ImageJ (National Institute of Health Image program v1.48 – manual/semi-automated segmentation)	Skeletal muscle index (SMI)	SMI independently predicted 30-day mortality in severely injured, mechanically ventilated trauma patients, while not correlating with ICU stay or ventilation duration
*Computed tomography abbreviated assessment of sarcopenia following trauma: The CAAST measurement predicts 6-month mortality in older adult trauma patients*	Leeper et al.	2016	445	All Trauma ICU (age > 65)	L3	iSite Picture Archiving and Communication System (PACS) – manual segmentation	Psoas muscle area computed tomography abbreviated assessment of sarcopenia following trauma (CAAST)	Low CAAST-defined psoas muscle area independently predicted 6-month post discharge mortality, with sarcopenia conferring nearly a fivefold increased risk of death.
*Computed Tomography Measurements of Sarcopenia Predict Length of Stay in Older Burn Patients*	Romanowski et al.	2021	83	Burns (age > 60)	T12 & L3	OsiriX MD version 9.5.1; (Pixmeo, Bernex, Switzerland) – semi-automated segmentation	Skeletal muscle index (SMI)	Lower paraspinal muscle SMI at T12 was significantly associated with prolonged hospital length of stay, whereas L3 muscle indices did not predict outcomes.
*Effect of sarcopenia on clinical and surgical outcome in elderly patients with proximal femur fractures*	Chang et al.	2018	91	Hip fractures (age > 65)	L4	General Electric Picture Archiving and Communicating System (S1000) – manual segmentation	Skeletal muscle index (SMI)	Lower SMI was independently associated with prolonged hospital stay, though it did not predict perioperative mortality or complications.
*Identification of Sarcopenia in Elderly Trauma patients: The Value of Clinical Competency and Experience*	Proksch et al.	2021	76	All Trauma ICU (age ≥ 50)	L4	Local Picture Archiving and Communication System (PACS) workstation – manual outlining	Psoas muscle index (PMI)	Sarcopenia was not associated with differences in short- or 6-month outcomes after trauma ICU admission.
*Impact of Pathologic Body Composition in Multiple Trauma Patients*	Poros et al.	2021	297	All Trauma ICU	L3	OsiriX (OsiriX Lite, Pixmeo, Geneva, Switzerland) – semi-automated segmentation	Total muscle area (TMA)	Low L3 total muscle area was not associated with ICU length of stay, duration of ventilation, sepsis, neurologic outcome, or mortality.
*Psoas cross-sectional area as a predictor of mortality and a diagnostic tool for sarcopenia in hip fracture patients*	Byun et al.	2019	494	Hip fractures (age > 50)	L4/5 intervertebral disc space	Aquarius iNtuition software, Version 4.4.12 (TeraRecon, Inc., San Mateo, CA, USA). – semi-automated segmentation	Psoas cross-sectional area (PCSA)	Decreased psoas cross-sectional area was significantly associated with higher 1-year mortality in hip fracture patients, particularly in women after adjustment for confounders.
*Psoas muscle area is associated with prognosis in elderly patients with hip fracture*	Byun et al.	2024	217	Female hip fractures (age > 50)	L4/5 intervertebral disc space	Aquarius iNtuition software, Version 4.4.12 (TeraRecon, Inc., San Mateo, CA, USA). – semi-automated segmentation	Psoas cross-sectional area (PCSA)	Low psoas CSA/BMI (lowest quartile) was independently associated with nearly double the risk of postoperative mortality and showed the strongest correlation with DXA-derived lean mass compared to other muscle groups
*Psoas Muscle Volume as an Indicator of Sarcopenia and Disposition in Traumatic Hip Fracture Patients*	Hovsepian et al.	2024	64	Hip fractures	T12 - Lesser trochanter of the femur	3D Slicer (Surgical Planning Laboratory, Brigham and Women’s Hospital, Boston, MA, USA) – semi-automatic segmentation	Total psoas volume (TPV)	Higher total psoas muscle volume was independently associated with increased likelihood of discharge home (vs. rehab facility) after surgically treated hip fracture, particularly in male patients.
*Sarcopenia and Frailty in Elderly Trauma Patients*	Fairchild et al.	2015	252	Blunt Trauma (Age > 65)	L4/5 intervertebral disc space	Slice-O-Matic (Tomovision, Montreal, QC, Canada) – manual/semi-automatic segmentation	Psoas cross-sectional area (PCSA)	Lower psoas CSA at L4–L5 was independently associated with loss of independence at discharge in elderly blunt trauma patients.
*Sarcopenia as a predictor of mortality in elderly blunt trauma patients: Comparing the masseter to the psoas using computed tomography*	Wallace et al.	2017	487	Blunt Trauma (Age > 65)	L4 and 2 cm below Zygomatic arch	Standard PACS software (*DR Systems Web Ambassador*,* version 8.2; Chicago*,* IL*,* USA*) – manual segmentation	Psoas cross-sectional area (PCSA).	Lower psoas cross-sectional area showed a non-significant trend toward higher 2-year mortality in elderly blunt trauma patients, with the fully adjusted model demonstrating a borderline protective effect of higher PCSA.
*Sarcopenia defined by a computed tomography estimate of the psoas muscle area does not predict frailty in geriatric trauma patients*	McCusker et al.	2019	325	All Trauma (age ≥ 65)	L3	Philips IntelliSpace Picture Archiving and Communication System (PACS; Philips Healthcare, Andover, MA, USA) – manual segmentation	Total Psoas Index - psoas muscle index (PMI) analogue	Low total psoas index at L3 was weakly correlated with frailty and independently predicted adverse discharge disposition, but not in-hospital mortality or complications in geriatric trauma patients.
Skeletal Muscle as a Factor Contributing to Better Stratification of Older Patients with Traumatic Brain Injury: A Retrospective Cohort Study	Shibahashi et al.	2017	74	Traumatic Brain Injury (age ≥ 60)	L3	SYNAPSE Picture Archiving and Communication System (PACS; Fujifilm Medical, Tokyo, Japan) – manual segmentation	Skeletal muscle cross-sectional area (CSA)	Reduced skeletal muscle area at L3 was independently associated with poor 6-month functional outcome after traumatic brain injury in older patients, with sarcopenia conferring a nearly fourfold increased risk of poor outcome.
*The psoas muscle index as a predictor of mortality and morbidity of geriatric trauma patients: experience of a major trauma center in Kobe*	Nishimura et al.	2020	405	All Trauma (age ≥ 65) & Injury Severity Scale (ISS) > 15	L3	Karos Health Picture Archiving and Communication System (PACS; Waterloo, ON, Canada) – manual segmentation	Psoas muscle index (PMI)	Low PMI was independently associated with higher in-hospital mortality and increased pneumonia risk in geriatric trauma patients, though 1-year survival and ADLs did not differ significantly.
*The psoas muscle index distribution and influence of outcomes in an Asian adult trauma population: an alternative indicator for sarcopenia of acute diseases*	Tee et al.	2021	939	All Trauma (age ≥ 16)	L4	Analytic Morphomics platform (Morphomic Analysis Group, University of Michigan, Ann Arbor, MI, USA) – semi-automatic	Psoas muscle index (PMI)	Extremely low PMI at L4 was independently associated with longer ICU stay in Asian trauma patients, though it did not significantly predict overall mortality or total hospital stay.
Association of Radiologic Indicators of Frailty With 1-Year Mortality in Older Trauma Patients 20Opportunistic Screening for Sarcopenia and Osteopenia	Kaplan et al.	2016	450	All Trauma in ICU (age ≥ 65)	L3	Slice-O-Matic, version 5.0 (TomoVision, Montreal, QC, Canada) – semi-automatic/manual segmentation	Skeletal muscle index (SMI)	Low Skeletal muscle index was independently associated with markedly increased 1-year mortality in older trauma ICU patients.
*The associations of psoas and masseter muscles with sarcopenia and related adverse outcomes in older trauma patients: a retrospective study*	Varma et al.	2022	204	≥ 65 Trauma	L4 and 2 cm below Zygomatic arch	Carestream Picture Archiving and Communication System (PACS; Carestream Health, Rochester, NY, USA) – manual segmentation	Psoas-lumbar vertebral index (PLVI)	Low PLVI (psoas-based sarcopenia) independently predicted higher in-hospital and 2-year mortality and reduced likelihood of discharge home in older trauma patients, whereas masseter CSA was not significantly associated with outcomes
*Decreased Lean Psoas Cross-Sectional Area Is Associated with Increased 1-Year All-Cause Mortality in Male Elderly Orthopaedic Trauma Patients*	Touban et al.	2019	558	Pelvic/Long bone fractures (age > 65)	L3/4 Intervertebral disc space	SliceOmatic soft-ware (Tomovision, Canada) – semi-automated segmentation	Lean psoas area (LPA) – Hybrid measure adjusted for fatty infiltration	Decreased lean psoas area was independently associated with increased 1-year all-cause mortality in elderly orthopaedic trauma patients, particularly in males.
*Lean psoas area does not correlate with clinical outcomes in moderately to severely injured older people*	Couch et al.	2017	205	All Trauma (age ≥ 65) & Injury Severity Scale (ISS) > 12	L4	Vitrea Advanced (Vital Images) – manual segmentation/outlining	Lean Psoas Area (LPA) – Hybrid measure adjusted for fatty infiltration	Lean psoas area was not an independent predictor of mortality, complications, or length of stay in moderately to severely injured older trauma patients.
The association of radiologic body composition parameters with clinical outcomes in level‑1 trauma patients	Sweet et al.	2023	404	All Trauma	L3	Quantib Body Composition, version 0.2.1 (Quantib, Rotterdam, The Netherlands) – automated segmentation	Psoas muscle index (PMI).	PMI was not independently associated with complications, pneumonia, delirium, or other adverse events, but lower PMI was linked to a higher likelihood of ICU admission and an unfavourable neurological outcome at discharge.
Computed tomography measured psoas density predicts outcomes in trauma	Yoo et al.	2017	151	Blunt Trauma (age ≥ 45)	L3	EasyViz Picture Archiving and Communication System (PACS; Karos Health, Waterloo, ON, Canada) – manual segmentation	Psoas muscle index (PMI)	Low psoas muscle index was an independent predictor of 90-day mortality only.

### Mortality

Across studies, reduced lumbar muscle quantity was commonly associated with increased short- and long-term mortality, although associations were not universal. The strongest and most consistent mortality signals were observed in older trauma populations and in critically injured cohorts, particularly when muscle quantity was normalised for body size rather than assessed as raw cross-sectional area. Mortality outcomes associated with lumbar muscle quantity AM are summarised in Table 2.

**Table 2:** Mortality data

AM Type	Author	Cohort (*n*)	Setting	AM metric	Outcome	Effect estimates	Interpretation
Lumbar quantity	Byun et al. (2019)	494	Hip fractures (age > 50)	(L4/5) PCSA	1-year mortality	HR 1.76, 95% CI 1.10–2.81, p = **0.017** (lowest vs. higher quintiles, women); no significant association in men	Low PCSA independently predicted higher 1-year mortality in women only
	Byun et al. (2024)	217	Female hip fractures (age > 50)	(L4/5) Psoas CSA/BMI (quartiles)	Postoperative mortality up to 5 years	HR 2.15, 95% CI 1.08–4.28, p = **0.029** (lowest quartile vs. others)	Lowest psoas CSA/BMI quartile independently predicted > 2-fold increased mortality risk
	Wallace et al. (2017)	487	Blunt trauma (age > 65)	(L4) PCSA	2-year mortality	HRs 0.57–0.64 (95% CI 0.39–0.82 to 0.43–0.95, *p* = 0.003–0.026) in partially adjusted models; Fully adjusted model: HR 0.68, 95% CI 0.46–1.00, *p* = 0.051	Lower PCSA associated with higher 2-year mortality; effect borderline in fully adjusted model
	Li et al. (2025)	334	Hip fracture post-surgery (two cohorts)	(T12) SMI	1-year mortality	OR 0.799, 95% CI 0.677–0.943, p = **0.008** (cohort 1); OR 0.881, 95% CI 0.784–0.991, p = **0.035** (cohort 2)	Each unit decrease in T12 SMI associated with ≈ 12–20% increased 1-year mortality risk
	Meyer et al. (2024)	472	Ventilated Trauma ICU patients	(L3) SMI	30-day mortality	HR 2.84, 95% CI 1.38–5.85, p = **0.004**	SMI-defined sarcopenia independently predicted higher 30-day mortality
	Kaplan et al. (2016)	450	All Trauma in ICU (age ≥ 65)	(L3) SMI	1-year mortality	HR 10.3, 95% CI 1.3–78.8, p = **0.030**	Sarcopenia markedly increased 1-year mortality risk
	Romanowski et al. (2021)	83	Burn (age > 60)	(T12 + L3) SMI	In-hospital mortality	T12 SMI: OR 0.694, 95% CI 0.194–2.487, *p* = 0.575; L3 SMI: OR 0.60, 95% CI 0.313–1.144, *p* = 0.120	No significant association between SMI and in-hospital mortality
	Chang et al. (2018)	91	Hip fractures (age > 65)	(L4) SMI	In-patient mortality	β = −0.001, 95% CI − 0.006 to 0.005, *p* = 0.852	SMI not associated with perioperative/in-patient mortality
	Nishimura et al. (2020)	405	All Trauma (age ≥ 65) & Injury Severity Scale (ISS) > 15	(L3) PMI	In-hospital mortality	OR 2.23, 95% CI 1.12–4.46, p = **0.023**	Low PMI independently predicted higher in-hospital mortality
	McCusker et al. (2019)	325	All Trauma (age ≥ 65)	(L3) Total psoas index (PMI analogue)	In-hospital mortality	Sarcopenia alone: OR 1.12, 95% CI 0.87–1.35, *p* = 0.730; Frailty and sarcopenia: OR 1.67, 95% CI 1.09–2.02, p = **0.030**	PMI-defined sarcopenia alone not predictive; combined frailty-sarcopenia increased mortality
	Tee et al. (2021)	939	All Trauma (age ≥ 16)	(L4) Extremely low PMI (ELPMI)	In-hospital mortality	Mortality 14.8% vs. 9.5% (ELPMI vs. non-ELPMI), *p* = 0.321; no adjusted mortality model	No significant mortality difference with ELPMI
	Proksch et al. (2021)	76	All Trauma ICU (age ≥ 50)	(L4) PMI	In-hospital, 3- and 6-month mortality	Similar mortality across sarcopenic vs. non-sarcopenic at all timepoints (all *p* > 0.300); no adjusted model	Sarcopenia not associated with short- or medium-term mortality
	Leeper et al. (2016)	445	All Trauma ICU (age > 65)	(L3) CAAST (PCSA indexed to body surface area)	6-month post-discharge mortality	HR 4.77, 95% CI 2.71–8.40, p < **0.001**	CAAST-defined sarcopenia was a strong independent predictor of 6-month mortality; not related to chronological age
	Tazerout et al. (2022)	114	All Trauma ICU (age ≥ 18)	(L3) ΔPMI (% decline = APTMA)	In-hospital mortality	Mortality 3%, 9%, 17% across low/moderate/severe APTMA, *p* = 0.200; no adjusted model	No significant association between acute PMI loss and mortality
	Couch et al. (2017)	205	All Trauma (age ≥ 65) & Injury Severity Scale (ISS) > 12	(L4) Lean psoas area (LPA)	In-hospital mortality	OR 1.000, 95% CI 0.998–1.002, *p* = 0.994	LPA not predictive of mortality
	Touban et al. (2019)	558	Pelvic/Long bone fractures (age > 65)	(L3/4) Lean psoas CSA	1-year mortality	Unadjusted: OR 0.93, 95% CI 0.90–0.96, p < **0.001**; Adjusted for age: HR 0.97, 95% CI 0.93–1.01, *p* = 0.106	Strong unadjusted association attenuated and became non-significant after age adjustment
	Yoo et al. (2017)	151	Blunt Trauma (age > 45)	(L3) PMI	90-day mortality	RR 5.95 95% CI 1.56–22.6; p = **0.008**	Patients classified as sarcopenic by PMI had a markedly higher mortality rate when compared to higher PMI quartiles.
	Varma et al. (2022)	204	All Trauma (age ≥ 65)	(L4) PLVI (psoas: vertebral index)	In-hospital mortality, 2-year survival	In-hospital mortality: OR 3.38, 95% CI 1.47–9.73, p = **0.006** 2-year survival: HR 1.90, 95% CI 1.11–3.25, p = **0.020**	Sarcopenia defined by PLVI was independently associated with increased in-hospital mortality and higher two-year mortality.
Lumbar Quality	Yoo et al. (2017)	151	Blunt Trauma (age > 45)	(L3) PD	90-day mortality	RR 5.95, 95% CI 1.56–22.6, p = **0.008**	Psoas density is an independent predictor of mortality
	Romanowski et al. (2021)	83	Burn (age > 60)	(T12 + L3) SMD	In-hospital mortality	T12 SMD: OR 1.52, 95% CI 0.79–2.92, *p* = 0.210 L3 SMD: OR 1.16, 95% CI 0.63–2.13, *p* = 0.640	Skeletal muscle density is not a predictor of mortality
	Chang et al. (2018)	91	Hip fractures (age > 65)	(L4) PSD	Perioperative mortality	β = 0.002, 95% CI − 0.001 to 0.004; *p* = 0.172	Paraspinal muscle density was not an independent predictor of mortality.
Adipose	Li et al. (2025)	334	Hip fracture post-surgery (two cohorts)	(T12) VSR, VFI, SFI	1-year mortality	Institution 1: T12 VFI OR 0.976, 95% CI 0.677–0.943, *p* = 0.063 T12 VSR OR 0.438, 85% CI 0.182–1.055, *p* = 0.066. Institution 2: T12 VSR OR 1.523, 95%CI 0.880–2.637, *p* = 0.133; T12 SFI 0.976, 95% CI 0.947–1.006, *p* = 0.120	Across two institutions, no independent association was demonstrated between CT-derived adiposity metrics (visceral fat index, subcutaneous fat index, or visceral-to-subcutaneous fat ratio) and mortality outcomes after multivariable adjustment.
Craniofacial	Wallace et al. (2017)	487	Blunt trauma (age > 65)	(2 cm below Zygomatic Arch) MCSA	2- year mortality	2-year mortality: HR 0.76, 95% CI 0.60–0.96, p = **0.023**	Lower masseter cross-sectional area independently predicted higher long-term mortality in an elderly trauma cohort.
	Varma et al. (2022)	204	All Trauma (age ≥ 65)	(2 cm below Zygomatic Arch) MCSA	In-hospital mortality, 2-year survival	In-hospital mortality: OR 1.18, 95% CI 0.56–5.05, *p* = 0.340 2-year survival: HR 1.76, 95% CI 0.94–3.31, *p* = 0.080	MCSA did not independently predict in-hospital mortality or long-term mortality.
	Hu et al. (2018)	108	Traumatic Brain Injury (age ≥ 55)	(2 cm below Zygomatic Arch) MCSA	30-day mortality	Multivariate Log Regression: Sarcopenia: OR 2.95, 95% CI 1.03–8.49; p = **0.045** MCSA: OR 0.66, 95% CI 0.46–0.95, p = **0.025** Cox Regression: Sarcopenia: HR 1.54, 95% CI 0.88–2.68, *p* = 0.140 MCSA: HR 0.78, 95% CI 0.62–0.97, p = **0.042**	In older patients with severe traumatic brain injury, CT-derived masseter sarcopenia was associated with increased 30-day mortality, while greater masseter muscle area conferred a protective effect, particularly when modelled as a continuous variable.
	Tanabe et al. (2019)	327	All Trauma (age ≥ 65)	(2 cm below Zygomatic Arch) MCSA, BAI	30-day mortality, 1-year mortality	30-day mortality: Analysed descriptively only (*p* = 0.140) 1-year mortality: MCSA: HR 2.0 (95% CI 1.2–3.1), p = **0.005** BAI: HR 2.0 (95% CI 1.1–3.5), p = **0.020** MCSA + BAI HR 3.4 95%CI 1.5–7.6; p = **0.002**	In older trauma patients, CT-derived masseter sarcopenia and brain atrophy were independently associated with increased 1-year mortality, while no association was observed with short-term (30-day) mortality. Patients with concurrent masseter sarcopenia and brain atrophy demonstrated substantially higher unadjusted 1-year mortality compared with those with neither condition, reflecting an additive accumulation of frailty-related risk.
Bone mineral density	Kaplan et al. (2016)	450	All Trauma in ICU (age ≥ 65)	(L3) BMD - Trabecular attenuation < 100HU	1-year mortality	Osteopenia: HR 11.9, 95% CI 1.3–107.4, p = **0.030** Sarcopenia + osteopenia adjusted HR 9.4,95% CI 1.2–75.4, p = **0.030**	CT-defined vertebral osteopenia, alone or in combination with sarcopenia, was independently associated with markedly increased 1-year mortality in older trauma patients.

Abbreviations: *AM* analytic morphomics, *APTMA* acute post-traumatic muscle atrophy, *BAI* brain atrophy index, *BMD* bone mineral density, *CAAST* cross-sectional area adjusted for stature, *CI* confidence interval, *CSA* cross-sectional area, *ELPMI* extremely low psoas muscle index, *HR* hazard ratio, *HU* Hounsfield units, *ISS* Injury Severity Score, *LPA* lean psoas area, *MCSA* masseter cross-sectional area, *NOS* Newcastle–Ottawa Scale, *OR* odds ratio, *PCSA* psoas cross-sectional area, *PD* psoas density, *PMI* psoas muscle index, *PSD* paraspinal muscle density, *RR* risk ratio, *SFI* subcutaneous fat index, *SMD* skeletal muscle density, *SMI* skeletal muscle index, *VAT/VFA* visceral adipose tissue/visceral fat area, *VFI* visceral fat index, *VSR* visceral-to-subcutaneous fat ratio

Effect estimates: Effect estimates are reported as adjusted values where multivariable models were available. Where adjusted analyses were not performed or not reported, unadjusted estimates or descriptive comparisons are presented and explicitly indicated. Effect estimates are reported with 95% confidence intervals (CI) and corresponding p values where available

Several studies demonstrated that lower psoas cross-sectional area (PCSA) predicted increased mortality. In large hip fracture cohorts studied by Byun et al., lower PCSA was independently associated with higher postoperative and longer-term mortality, with sex-specific effects observed in women [21, 22]. In these cohorts, patients in the lowest psoas area categories had significantly increased mortality risk (HR 1.76, 95% CI 1.10–2.81, *p* = 0.017; HR 2.15, 95% CI 1.08–4.28, *p* = 0.029). Similar associations were reported in elderly blunt trauma populations by Wallace et al., although the strength of association lessened with increasing model adjustment (HR 0.68, 95% CI 0.46–1.00, *p* = 0.051) [23].

Indices adjusting muscle area for body size, such as skeletal muscle index (SMI) and psoas muscle index (PMI), demonstrated more consistent prognostic value. Lower SMI independently predicted mortality in multiple cohorts, including older trauma patients and critically ill populations. For example, Li et al. reported that reduced T12 SMI independently predicted 1-year mortality across two cohorts (OR 0.799, 95% CI 0.677–0.943, *p* = 0.008; OR 0.881, 95% CI 0.784–0.991, *p* = 0.035) [24]. In mechanically ventilated trauma patients, Meyer et al. showed SMI-defined sarcopenia was associated with substantially increased 30-day mortality (HR 2.84, 95% CI 1.38–5.85, *p* = 0.004) [25], while Kaplan et al. observed a marked increase in 1-year mortality among sarcopenic ICU trauma patients (HR 10.3, 95% CI 1.3–78.8, *p* = 0.030) [26].

However, two studies found no significant associations between SMI and mortality. Romanowski et al. included 83 burns patients, all aged > 60, and found no association with SMI and in-patient mortality on two vertebral levels measured (T12 and L3) [27]. Similar findings were reported by Chang et al. who found no significant associations between low SMI and perioperative (in-hospital) mortality in 91 older hip fracture patients [28].

PMI-based findings were more heterogeneous. Some studies demonstrated strong mortality associations, including increased in-hospital mortality among elderly trauma patients with low PMI studied by Nishimura et al. (OR 2.23, 95% CI 1.12–4.46, *p* = 0.023) [29]; and markedly higher 90-day mortality among sarcopenic patients investigated by Yoo et al. (RR 5.95, 95% CI 1.56–22.6, *p* = 0.008) [30]. McCusker et al., in a large cohort of older trauma patients (*n* = 325), found PMI to not be an independent predictor of mortality alone, but combined with frailty–measured by the Trauma-Specific Frailty Index (TSFI)–sarcopenia and frailty significantly predicted in-patient mortality (OR 1.67, 95% CI 1.09–2.02, *p* = 0.030) [31].

Two studies did not find an association between PMI and mortality. Proksch et al., in a cohort of 76 ICU trauma patients (age > 50) found no associations between sarcopenic patients (classified by low PMI) and in-hospital, 3-month, or 6-month mortality [32]. Similarly, Tee et al. found no differences in in-hospital mortality between patients with an extremely low PMI (ELMPI)–two standard deviations below sex-specific mean–and a non-ELMPI cohort [33].

Couch et al. and Touban et al. combined PCSA with Hounsfield units to create a hybrid measurement producing a lean psoas area (adjusted for fatty infiltration of the muscle), but found no significant predictive associations with mortality [34, 35].

A notable large-scale contribution was the multicentre CAAST study by Leeper et al., where sarcopenia defined using the CT Abbreviated Assessment of Sarcopenia after Trauma was the strongest independent predictor of 6-month post-discharge mortality (HR 4.77, 95% CI 2.71–8.40, *p* < 0.001), independent of chronological age [36].

Two novel measurements were suggested by Varma et al. and Tazerout et al. Varma et al. suggested not adjusting the PCSA by height, but rather by dividing it through the vertebral body area to create a psoas: vertebral index (PLVI). This measurement independently predicted in-hospital mortality (OR 3.38, 95% CI 1.47–9.73, *p* = 0.006) and 2-year mortality (HR 1.90, 95% CI 1.11–3.25, *p* = 0.020) [37]. Tazerout et al. who suggested using a change in PMI (ΔPMI) measured on two occasions in adult ICU trauma patients but found no significant associations with in-hospital mortality [38].

### Hospital and ICU length of stay

Associations between muscle quantity and LOS were less consistent than those observed for mortality. Table 3 summarises LOS outcomes reported for lumbar muscle quantity AM. Several studies demonstrated increased hospital or ICU LOS among patients with reduced muscle quantity, although null findings were also common.

**Table 3:** Length of stay data

AM Type	Study	Cohort (*n*)	Setting	Metric	LOS outcome	Effect estimates	Interpretation
Lumbar Quantity	Romanowski et al. (2021)	83	Burn (age > 60)	(T12) SMI; paraspinal CSA	Hospital LOS	T12 SMI: β = −9.21, 95% CI − 15.72 to − 2.70, p = **0.007**; T12 CSA: β − 7.38, 95% CI − 14.08 to − 0.68, p = **0.032**; L3: No association	Lower T12 muscle mass independently associated with longer hospital stays; L3 metrics non-significant
	Chang et al. (2018)	91	Hip fractures (age > 65)	(L4) SMI	Hospital LOS	β = −0.140, 95% CI − 0.268 to − 0.013, p = **0.032**	Lower SMI independently predicted longer hospital LOS
	Meyer et al. (2024)	472	Ventilated Trauma ICU patients	(L3) SMI	ICU LOS	Univariable analysis: β = 0.88, 95% CI − 0.56 to 2.32, *p* = 0.233; SMI not included in multivariable model	No significant association between SMI and ICU LOS
	Sweet et al. (2023)	404	All Trauma (no severe TBI)	(L3) PMI	Hospital LOS; ICU LOS; duration of mechanical ventilation	Hospital LOS: β = 0.97, 95% CI 0.92 to 1.02, *p* = 0.210; ICU LOS: β = 0.94, 95% CI 0.80 to 1.11, *p* = 0.490; Ventilation: β = 0.89, 95% CI 0.79 to 1.01, *p* = 0.800	PMI no independently associated with LOS or DMV
	Tee et al. (2021)	939	All Trauma (age ≥ 16)	(L4) Extremely low PMI (ELPMI)	ICU LOS; hospital LOS	ICU LOS: β = 3.881, 95% CI 0.878 to 6.884, p = **0.011**; Hospital LOS: 24.5 vs. 19.7 days (ELPMI vs. non-ELPMI), *p* = 0.133	ELPMI independently predicted prolonged ICU stay; hospital LOS difference not significant
	Tazerout et al. (2022)	114	All Trauma ICU (age ≥ 18)	(L3) ΔPMI (% decline)	ICU LOS; hospital LOS	ICU LOS: β = 0.91, 95% CI 0.66 to 1.15, p < **0.001**; Hospital LOS: β 0.63, 95% CI 0.31 to 0.96, p < **0.001**	Each 1% decrease in PMI independently associated with longer ICU and hospital LOS
	Couch et al. (2017)	205	All Trauma (age ≥ 65) & Injury Severity Scale (ISS) > 12	(L4) LPA	Hospital LOS	Spearman’s ρ, *p* = 0.648; no adjusted model	No correlation between LPA and hospital LOS
	Poros et al. (2021)	297	All Trauma ICU (age ≥ 18)	(L3) Total muscle area (TMA)	Duration of mechanical ventilation; ICU LOS	Ventilation: β = 0.005, 95% CI 0.000 to 0.010, *p* = 0.063; ICU LOS: β 0.0007, 95% CI − 0.002 to 0.004, *p* = 0.663	TMA not independently associated with ventilation duration or ICU LOS (borderline trend for ventilation only)
	Varma et al. (2022)	204	All Trauma (age ≥ 65)	(L4) PLVI (psoas: vertebral index)	Hospital LOS	OR 1.21, 95% CI 0.85–1.71, *p* = 0.290	PLVI-defined sarcopenia not associated with hospital LOS
	Yoo et al. (2017)	151	Blunt Trauma (age > 45)	L3 (PMI)	Mean LOS and Prolonged LOS (LOS ≥ 7 days)	RR 0.78, 95% CI 0.33–1.41, *p* = 0.402	PMI was not associated with hospital LOS; no ICU LOS outcomes were reported.
Lumbar Quality	Yoo et al. (2017)	151	Blunt Trauma (age > 45)	L3 (PD)	Mean LOS and Prolonged LOS (LOS ≥ 7 days)	RR 1.63 (95% CI 1.02–2.59), *p* = 0.048	Sarcopenic patients with low psoas density had significantly higher odds of prolonged hospital length of stay
	Romanowski et al. (2021)	83	Burn (age > 60)	(T12 + L3) SMD	Hospital LOS	T12 SMD: β = 0.71 days per HU (*p* = 0.840) L3 SMD: β = −2.22 days per HU (*p* = 0.448)	No association found between skeletal muscle density and LOS.
	Chang et al. (2018)	91	Hip fractures (age > 65)	(L4) PSD	Hospital LOS	β = −0.073 days per HU 95% CI − 0.126 to − 0.020, p = **0.008**	Paraspinal Density is a strong independent predictor of prolonged LOS.
	Sweet et al. (2023)	404	All Trauma (no severe TBI)	(L3) PMRA	Hospital LOS; ICU LOS; duration of mechanical ventilation	Hospital LOS: β = 0.84, 95% CI 0.76–0.93; p = **0.001**; ICU LOS: β = 0.80, 95% CI 0.65–0.99; p = **0.040**; Ventilation: β = 0.77, 95% CI 0.66–0.90; p = **0.002**	Muscle density is a robust predictor of inpatient resource utilisation across multiple domains, outperforming muscle-quantity measures in the same cohort.
Adipose	Sweet et al. (2023)	404	All Trauma (no severe TBI)	(L3) VFA	Hospital LOS; ICU LOS; duration of mechanical ventilation	Hospital LOS: β 1.02, 95% CI 0.93,1.12, *p* = 0.690 ICU LOS: β 1.07, 95%CI 0.81,1.43; *p* = 0.620 Ventilation: β 1.06, 95% CI 0.86,1.31; *p* = 0.550	Visceral fat area was not independently associated with Hospital LOS, ICU LOS or duration of mechanical ventilation.
	Poros et al. (2021)	297	All Trauma ICU (age ≥ 18)	(L3) SAT, VAT	Duration of mechanical ventilation; ICU LOS	ICU LOS: SAT: β = −0.0005, 95% CI − 0.002 to 0.001; *p* = 0.355 VAT: β = −0.0005, 95% CI − 0.002 to 0.001; *p* = 0.453 Ventilation: SAT: β = −2.5 × 10⁻⁵, 95% CI − 0.002 to 0.002; *p* = 0.981 VAT: β = 0.001, 95% CI − 0.002 to 0.004; *p* = 0.415	Subcutaneous Adipose Tissue and Visceral Adipose Tissue were not independently associated with prolonged ICU LOS or duration of mechanical ventilation.
	Docimo et al. (2015)	57	All Trauma (age ≥ 18) undergoing abdominal surgery	(L4/5) VSR, SFA, VFA, PNF	Hospital LOS	Hospital LOS: VSR (VSR ≥ 0.4 vs. < 0.4, *p* = 0.380) unadjusted PNF correlated with LOS (unadjusted; *p* = 0.031)	Perinephric fat thickness was associated with longer LOS on unadjusted analysis, while other adiposity metrics showed no association with LOS.
Craniofacial	Lisiecki et al. (2013)	16	Mandible Fracture	(Plane through external auditory meatus, superior orbital rim and mandibular coronoid process) TMT, ZBT, TLMT	Hospital LOS, ICU LOS	Hospital LOS: ZBT: *r* = −0.6495; p < **0.001** TMT: *r* = −0.3698; p = **0.026** TLMT: *r* = −0.3776; p = **0.023** ICU LOS: ZBT: *r* = −0.5742; p < **0.001**	Reduced zygomatic bone thickness and temporalis muscle thickness were associated with longer hospital and intensive care unit stays, with the strongest correlation observed between zygomatic bone thickness and hospital length of stay.
	Wallace et al. (2017)	487	Blunt trauma (age > 65)	(2 cm below Zygomatic Arch) MCSA	Hospital LOS, ICU LOS	Hospital LOS: (no p values reported) MCSA: ρ = −0.098 (*p* > 0.050) ICU LOS: MCSA: ρ = −0.037 (*p* > 0.050)	Craniofacial sarcopenia, measured by masseter muscle area, was not associated with hospital or ICU length of stay
	Varma et al. (2022)	204	All Trauma (age ≥ 65)	(2 cm below Zygomatic Arch) MCSA	Hospital LOS	Hospital LOS: OR 1.07 (95% CI 0.73–1.59), *p* = 0.720	MCSA sarcopenia was not associated with hospital length of stay in adjusted analyses of older trauma patients
	Hu et al. (2018)	108	Traumatic Brain Injury (age ≥ 55)	(2 cm below Zygomatic Arch) MCSA	Hospital LOS, ICU LOS, Hospital free days, ICU free days	Hospital LOS: 19.7 ± 36.4 vs. 10.9 ± 16.2 days (*p* = 0.110) ICU LOS: ICU LOS—15.3 ± 36.4 vs. 8.6 ± 12.7 days (*p* = 0.350) Hospital-free days (30 days)—0.2 ± 1.0 vs. 3.5 ± 7.0 (p < **0.001**) ICU-free days—2.4 ± 7.6 vs. 4.2 ± 7.1 (*p* = 0.290)	Although sarcopenic patients demonstrated longer hospital and ICU stays, only hospital-free days were significantly reduced, likely reflecting higher early mortality rather than prolonged resource utilisation.
Bone mineral density	Armstrong et al. (2022)	336	Motor Vehicle Collision (age ≥ 50)	(L3) SMI/BMD -	Hospital LOS, ICU LOS	Hospital LOS: β = −0.172, 95% CI − 0.418 to 0.074, *p* = 0.169 ICU LOS: OR = 0.788, 95% CI 0.393–1.580, *p* = 0.503	Osteosarcopenia was not independently associated with increased hospital or ICU LOS
	Kaplan et al. (2016)	450	All Trauma in ICU (age ≥ 65)	(L3) BMD - Trabecular attenuation < 100HU	Hospital LOS, ICU LOS	Hospital LOS: No significance between groups (*p* = 0.990) ICU LOS: No significance between groups (*p* = 0.450) No further modelling attempted.	CT-derived vertebral osteopenia was not associated with increased hospital or ICU length of stay in older trauma patients.

Abbreviations: *AM* analytic morphomics, *APTMA* acute post-traumatic muscle atrophy, *BMD* bone mineral density, *CI* confidence interval, *CSA* cross-sectional area, *ELPMI* extremely low psoas muscle index, *HR* hazard ratio, *HU* Hounsfield units, *ICU* intensive care unit, *ISS* Injury Severity Score, *LOS* length of stay, *LPA* lean psoas area, *MCSA* masseter cross-sectional area, *OR* odds ratio, *PCSA* psoas cross-sectional area, *PD* psoas density, *PMI* psoas muscle index, *PMRA* psoas muscle radiation attenuation, *PSD* paraspinal muscle density, *RR* risk ratio, *SAT* subcutaneous adipose tissue, *SFA* subcutaneous fat area, *SMD* skeletal muscle density, *SMI* skeletal muscle index, *TMA* total muscle area, *VAT/VFA* visceral adipose tissue/visceral fat area, *VSR* visceral-to-subcutaneous fat ratio, *ZBT* zygomatic bone thickness, *TMT* temporalis muscle thickness, *TLMT* temporalis–lateral muscle thickness

Effect estimates: Effect estimates are reported as adjusted values where multivariable models were available. Where adjusted analyses were not performed or not reported, unadjusted estimates, correlation coefficients, or descriptive comparisons are presented and explicitly indicated. Regression coefficients (β) represent the change in length of stay (days) per unit change in the predictor unless otherwise specified. Effect estimates are reported with 95% confidence intervals (CI) and corresponding p values where available

In selected cohorts, lower SMI independently predicted longer hospital or ICU stays. Romanowski et al. demonstrated that reduced paraspinal muscle mass at T12 was independently associated with prolonged hospital LOS in trauma burn patients (β = −9.21, 95% CI − 15.72 to − 2.70, *p* = 0.007) [27], while Chang et al. reported lower SMI predicted longer hospital stay in elderly hip fracture patients (β = −0.140, 95% CI − 0.268 to − 0.013, *p* = 0.032) [28]. Similar findings were reported by Tee et al. who found that ELMPI independently predicted prolonged ICU stay (β = 3.881, 95% CI 0.878–6.884, *p* = 0.011) [39].

Conversely, several studies (Meyer et al., Sweet et al., Yoo et al., Poros et al., Varma et al., Couch et al.) reported no independent association between static muscle quantity measures and LOS, particularly in mechanically ventilated trauma cohorts and mixed-severity populations [25, 30, 34, 40, 41].

However, a study assessing dynamic muscle change demonstrated stronger associations. Tazerout et al. showed that acute post-traumatic muscle atrophy (ΔPMI) independently predicted longer ICU and hospital stays, with each 1% decrease in PMI associated with increased ICU LOS (β = 0.91, 95% CI 0.66 to 1.15, *p* < 0.001) and hospital LOS (β = 0.63, 95% CI 0.31 to 0.96, *p* < 0.001) [38]. These findings suggest that evolving muscle loss during critical illness may better capture LOS risk than baseline sarcopenia alone.

### Adverse outcomes and complications

Throughout lumbar quantity AM studies, reduced muscle quantity showed more consistent associations with functional adverse outcomes than with broad composite complications. Table 5 summarises complication and adverse outcome measures reported for lumbar muscle quantity AM. Lower psoas muscle area was a strong independent predictor of unfavourable discharge disposition in elderly trauma patients, with Fairchild et al. reporting that each 1 cm² increase in psoas CSA reduced the odds of dependent discharge (OR 0.836, 95% CI 0.772–0.905, *p* < 0.001). Similarly, McCusker et al. found sarcopenia predicted unfavourable discharge (OR 1.41, 95% CI 1.04–1.87, *p* < 0.050), with stronger associations when sarcopenia coexisted with frailty (OR 2.48, 95% CI 1.79–2.97, *p* < 0.050) [31]. This aligned with findings by Hovsepian et al. who found that higher total psoas volume (TPV) independently predicted favourable discharge in men following a hip fracture (OR 1.014, 95% CI 1.001–1.026, *p* = 0.028) [42].

Neurologic outcomes also demonstrated consistent associations. In mixed trauma cohorts (Sweet et al.), lower PMI predicted unfavourable neurologic outcome (OR 0.62, 95% CI 0.45–0.85, *p* = 0.003) [40], while in older adults with traumatic brain injury (Shibahashi et al.), sarcopenia was independently associated with poor 6-month functional outcome (OR 3.88, 95% CI 1.14–13.2, *p* = 0.031) [43].

In contrast, associations between muscle quantity and composite in-hospital complications were inconsistent. Several studies (Sweet et al., Couch et al., Poros et al., Yoo et al.) reported no independent relationship between sarcopenia and overall complication risk [30, 34, 37, 40, 41]. However, selected complications showed significant associations in specific populations. PMI-defined sarcopenia (Nishimura et al.) was associated with pneumonia in geriatric trauma patients (OR 2.16, 95% CI 1.25–3.72, *p* < 0.010) [29], while acute muscle atrophy (Tazerout et al.) strongly predicted infectious complications and prolonged organ-support requirements in ICU trauma patients [38].

## Lumbar muscle quality analytic morphomics

CT-derived muscle quality metrics quantify tissue composition using attenuation values in Hounsfield Units (HU), reflecting fatty infiltration rather than muscle quantity alone. From this section, muscle quality measures demonstrated more consistent associations with clinical outcomes than muscle quantity metrics, particularly for length of stay and adverse functional outcomes Table 4.

**Table 4:** Lumbar muscle quality analytic morphomics

Study Name	Author	Date	Cohort (*n*)	Population	Level Measured	Software	Analytic Morphomic Variables	Conclusion
The association of radiologic body composition parameters with clinical outcomes in level‑1 trauma patients	Sweet et al.	2023	404	All Trauma	L3	Quantib Body Composition, version 0.2.1 (Quantib, Rotterdam, The Netherlands) – automated segmentation	Psoas muscle radiation attenuation (PMRA) – mean Hounsfield units	Lower psoas muscle radiation attenuation (a marker of poor muscle quality/fatty infiltration) was independently associated with more complications, pneumonia, delirium, longer ICU and hospital stays, and worse functional outcomes.
2017	Yoo et al.	2017	151	Blunt Trauma (age ≥ 45)	L3	EasyViz Picture Archiving and Communication System (PACS; Karos Health, Waterloo, ON, Canada) – manual segmentation	Psoas density (PD) – area weighted mean attenuation. (HUAC)	Low psoas muscle density was a stronger predictor of adverse outcomes—including 90-day mortality, complications, prolonged hospital stay, and dependent discharge—than psoas muscle area.
Pre-injury sarcopenia and the association with discharge destination in critical care trauma patients	Badminton et al.	2025	197	All Trauma ICU (age ≥ 16)	L4	Slice-O-Matic, version 5.0 (TomoVision, Montreal, QC, Canada). – semi-automated/manual segmentation	Psoas muscle density (PMD) - area weighted mean attenuation. (HUAC)	Lower psoas muscle density (PMD, a marker of poor muscle quality) was associated with a significantly reduced likelihood of independent (home) discharge, independent of age, frailty, and injury severity.
Computed Tomography Measurements of Sarcopenia Predict Length of Stay in Older Burn Patients	Romanowski et al.	2021	83	Burns (age > 60)	T12 & L3	OsiriX MD version 9.5.1; (Pixmeo, Bernex, Switzerland) – semi-automated segmentation	Skeletal muscle density (SMD) – mean Hounsfield units	Skeletal muscle density was not predictive of LOS or mortality; LOS associations were observed for muscle quantity only.
Effect of Sarcopenia on clinical and surgical outcome in elderly patients with proximal femur fractures	Chang et al.	2018	91	Hip fractures (age ≥ 65)	L4	OsiriX version 8.0.2 (Pixmeo SARL, Geneva, Switzerland) – semi-automated/manual segmentation	Paraspinal muscle density (PSD) - mean Hounsfield units	Lower paraspinal muscle density (PSD) on CT was independently associated with prolonged hospital stay and higher perioperative blood transfusion requirements in elderly patients with proximal femur fractures

### Mortality

Associations between muscle quality and mortality were heterogeneous with mortality outcomes for lumbar quality AM detailed in Table 2. In a blunt trauma cohort of 151 patients over the age of 45, Yoo et al. reported a strong association between low psoas muscle density and short-term mortality, with patients in the lowest attenuation quartile experiencing markedly higher 90-day mortality compared with those with preserved density (RR 5.95; 95% CI 1.56–22.6; *p* = 0.008) [30].

In contrast, two cohorts demonstrated no mortality association. Romanowski et al. found no relationship between skeletal muscle density at either T12 or L3 and in-hospital mortality in 83 older trauma burn patients [27], while Chang et al. similarly reported no association between paraspinal muscle density and per-operative mortality in 91 older hip fracture patients after multivariable adjustment [28]. These findings suggest that muscle quality may predict mortality primarily in mixed blunt trauma populations, with attenuated effects in more homogeneous or procedure-specific cohorts.

### Hospital and ICU length of stay

Muscle quality measures showed more consistent associations with LOS than with mortality, although effect sizes varied across studies. Table 3 summarises LOS outcomes reported for lumbar muscle quality AM. Lower psoas density was associated with an increased risk of prolonged hospitalisation in blunt trauma patients (LOS ≥ 7 days) in Yoo et al. (RR 1.63; 95% CI 1.02–2.59; *p* = 0.048) [30]. Similarly, Chang et al. demonstrated that lower paraspinal muscle density independently predicted longer hospital stays in elderly hip fracture patients, with each unit decrease in density associated with incremental prolongation of admission (β = −0.073 days per HU 95% CI − 0.126 to − 0.020, *p* = 0.008) [28]. The strongest and most consistent LOS effects were reported by Sweet et al., where higher psoas radiation attenuation independently predicted shorter hospital LOS (β = 0.84, 95% CI 0.76 to 0.93; *p* = 0.001), ICU LOS (β = 0.80, 95% CI 0.65 to 0.99; *p* = 0.040), and duration of mechanical ventilation (β = 0.77, 95% CI 0.66 to 0.90; *p* = 0.002) [40]. These findings indicate that attenuation-based metrics, particularly when modelled continuously, provide clinically relevant prognostic information for resource utilisation.

The only study to contrast the findings above was by Romanowski et al. who found no association between skeletal muscle density and LOS on T12 and L3 measurements [27]. This null association was observed in a highly specific burn population, and contrasts with the more consistent relationship between muscle attenuation and length of stay reported across heterogenous trauma cohorts.

### Adverse outcomes and complications

Reduced muscle quality was more consistently associated with adverse functional outcomes and selected complications. Table 5 summarises complication and adverse outcome measures reported for lumbar muscle quality AM. In Yoo et al., sarcopenia defined by low psoas density was associated with substantially higher risks of in-hospital complications (RR 2.30, 95% CI 1.37–3.85; *p* = 0.002) and dependent discharge (RR 2.14, 95% CI 1.18–3.88; *p* = 0.015) [30]. Similarly, Sweet et al. demonstrated that higher muscle attenuation was independently protective against complications, delirium, ICU admission, and unfavourable neurologic outcome, with consistent effects across outcomes (all *p* < 0.050) [40]. In a critical-care trauma cohort, Badminton et al. reported no association between pre-injury muscle density and complications or ICU resource use; however, sarcopenic patients were significantly less likely to achieve independent discharge (OR 0.24; 95% CI 0.06–0.89; *p* = 0.030) [44].

**Table 5:** Complications and adverse outcomes data

AM Type	Study	Cohort	Setting	Metric	Outcome	Effect estimate	Interpretation
Lumbar Quantity	Fairchild et al. (2015)	252	Blunt Trauma (age. 65)	(L4/5) PCSA	Discharge disposition (dependent vs. independent)	OR 0.836 per 1 cm² increase, 95% CI 0.772–0.905, p < **0.001**	Higher PCSA strongly associated with reduced odds of dependent discharge
	McCusker et al. (2019)	325	All Trauma (age ≥ 65)	(L3) Total psoas index (PMI analogue)	In-hospital complications; discharge disposition	Sarcopenia alone: Complications OR 1.21, 95% CI 0.91–1.50; Adverse discharge OR 1.41, 95% CI 1.04–1.87. Frailty + sarcopenia: Complications OR 2.03, 95% CI 1.12–2.83; Adverse discharge OR 2.48, 95% CI 1.79–2.97	Sarcopenia associated with unfavourable discharge; combined frailty–sarcopenia predicted both complications and adverse discharge more strongly.
	Sweet et al. (2023)	404	All Trauma (no severe TBI)	(L3) PMI	Composite complications; pneumonia; delirium; ICU admission; neurologic outcome (GOS 1–3)	Any complication: OR 0.92, 95% CI 0.76–1.12, *p* = 0.430; pneumonia: OR 0.91, 95% CI 0.71–1.18, *p* = 0.490; delirium: OR 0.92, 95% CI 0.63–1.33, *p* = 0.660; ICU admission: OR 0.79, 95% CI 0.65–0.95, p = **0.011**; Unfavourable GOS: OR 0.62, 95% CI 0.45–0.85, p = **0.003**	PMI not associated with complications, pneumonia, or delirium, but lower PMI independently predicted ICU admission and poor neurologic outcome
	Shibahashi et al. (2017)	74	Traumatic Brain Injury (age ≥ 60)	(L3) Skeletal muscle CSA	Poor 6-month functional outcome (GOS 1–2)	OR 3.88, 95% CI 1.14–13.2, p = **0.031**	Sarcopenia associated with nearly fourfold higher odds of poor neurological outcome
	Hovsepian et al. (2024)	64	Hip Fracture	Total psoas volume (TPV)	Discharge home vs. rehabilitation; ICU admission	Men: OR 1.014, 95% CI 1.001–1.026, p = **0.028** for discharge home; no association in women (no regression model); TPV not associated with ICU admission	Higher TPV independently predicted favourable discharge in men only; no ICU association
	Tazerout et al. (2022)	114	All Trauma ICU (age ≥ 18)	(L3) ΔPMI (% decline)	Pneumonia; other infections; thromboembolic events; RRT; duration of MV; catecholamine use	Pneumonia p = **0.006**; other infections p = **0.014**; Thromboembolic events *p* = 0.041; RRT *p* = 0.056. Ventilation: β = 0.78 days per 1% ΔPMI, 95% CI 0.61 to 0.95, p < **0.001**; Catecholamine use: β = 0.23 days, 95% CI 0.13 = 0.33, p < **0.001**	Greater acute PMI loss associated with higher rates of pneumonia, infection, thromboembolism and prolonged organ-support requirements
	Nishimura et al. (2020)	405	All Trauma (age ≥ 65) & Injury Severity Scale (ISS) > 15	(L3) PMI	Pneumonia; UTI; VTE/PE; discharge disposition	Pneumonia: OR 2.16, 95% CI 1.25–3.72, p < **0.010**; UTI: OR 1.16, 95% CI 0.63–2.14, *p* = 0.630; VTE/PE: OR 1.30, 95% CI 0.79–2.12, *p* = 0.310; Discharge outcomes not significant	Low PMI significantly increased pneumonia risk; other complications and discharge not associated
	Poros et al. (2021)	297	All Trauma ICU (age ≥ 18)	(L3) TMA	Sepsis; shock on admission; neurological outcome; MV duration; ICU therapy duration	Sepsis: OR 1.0069, 95% CI 0.999–1.016, *p* = 0.098; Shock: OR 0.999, 95% CI 0.991–1.008, *p* = 0.921; GOS: β = 0.0002, 95% CI − 0.003 to 0.003, *p* = 0.886; MV duration: β = 0.005, 95% CI 0.000 to 0.010, *p* = 0.063; ICU therapy: β = 0.0007, 95% CI − 0.002 to 0.004, *p* = 0.663	TMA-defined sarcopenia not independently associated with sepsis, shock, neurological outcome, MV duration, or ICU therapy duration
	Couch et al. (2017)	205	All Trauma (age ≥ 65) & Injury Severity Scale (ISS) > 12	(L4) LPA	Inpatient complications (respiratory + VTE)	OR 1.000, 95% CI 0.998–1.002, *p* = 0.963	LPA not predictive of inpatient complications
	Yoo et al. (2017)	151	Blunt Trauma (age > 45)	(L3) PMI	Complications; Dependent discharge	Complications: RR 1.03, 95% CI 0.56–1.92, *p* = 0.915; Dependent discharge: RR 0.86, 95% CI 0.18–1.25, *p* = 0.102	PMI was not associated with complications or discharge disposition.
	Varma et al. (2022)	204	All Trauma (age ≥ 65)	(L4) PLVI (psoas: vertebral index)	Any Complication	OR 1.08, 95% CI 0.52–2.21, *p* = 0.840	No independently significant association between PLVI and any complications.
Lumbar Quality	Yoo et al. (2017)	151	Blunt Trauma (age > 45)	L3 (PD)	In-hospital complications; dependent discharge	Complications: RR 2.30, 95% CI 1.37–3.85; p = **0.002**, Dependent discharge: RR 2.14, 95% CI 1.18–3.88; p = **0.015**	Low Psoas Density is associated with greater risk of in-hospital complications and chance of dependent discharge
	Sweet et al. (2023)	404	All Trauma (no severe TBI)	(L3) PMRA	Complication, Delirium, Infection, Unfavoural GOS, ICU admission	Complications: OR 0.60, 95% CI 0.42–0.85; p = **0.004**, Delirium: OR 0.49, 95% CI 0.28–0.87, p = **0.014**, Infection: OR 0.65, 95% CI 0.47–0.94, *p* = 0.720, Unfavourable GOS: OR 0.570, 95% CI 0.36–0.92, p = **0.021**; ICU admission: OR 0.54, 95% CI 0.38–0.77, p = **0.001**	Psoas radiation attenuation significant predictor of complications and adverse outcomes.
	Chang et al. (2018)	91	Hip fractures (age > 65)	(L4) PD	Medical Complication, Blood Transfusion, Readmission	Medical Complication - β = 0.003 95% CI − 0.002 to 0.008, *p* = 0.255; Blood Transfusion: β = − 0.055, 95% CI − 0.093 to − 0.018, p = **0**.**004**. Readmission: β = − 0.002, 95% CI − 0.007 to 0.003, *p* = 0.365	Paraspinal density does not predict medical complications or readmission, but does predict blood transfusion required.
	Badminton et al. (2025)	197	All Trauma ICU (age ≥ 16)	(L4) PMD	Independent discharge	Independent Discharge: OR 0.30, 95% CI 0.17–0.81; p = **0.010**	Sarcopenia based on Psoas Muscle Density is an independent predictor of independent discharge, especially in patients over the age of 65.
Adipose	Sweet et al. (2023)	404	All Trauma (no severe TBI)	(L3) VFA	Any Complication, Infection (All), Pneumonia, UTI, Wound infection, Delirium, Unfavourable GOS.	Any complication: OR 1.28, 95% CI 0.92–1.77; *p* = 0.150 Infection: OR 1.30, 95% CI 0.92–1.83; *p* = 0.140 Pneumonia: OR 1.46, 95% CI 0.96–2.23; *p* = 0.078 UTI: OR 0.65, 95% CI 0.28–1.53; *p* = 0.320 Wound infection: OR 1.18, 95% CI 0.58–2.39; *p* = 0.650 Delirium: OR 1.95, 95% CI 1.12–3.41; p = **0.018** Unfavourable GOS: OR 1.44, 95% CI 0.90–2.29; *p* = 0.130	VFA was predictive of Delirium but does not independently predict other complications or outcomes.
	Poros et al. (2021)	297	All Trauma ICU (age ≥ 18)	(L3) SAT, VAT	Sepsis, Poor GOS	Sepsis VAT: OR 1.001, 95% CI 0.997–1.005, *p* = 0.496 SAT: OR 0.998, 95% CI 0.996–1.002, *p* = 0.485 Poor GOS (1–2) VAT: β = −0.0002, 95% CI − 0.002 to 0.001, *p* = 0.749 SAT: β = 2.96 × 10⁻⁵, 95% CI − 0.001 to 0.001, *p* = 0.951	Subcutaneous or visceral fat was not associated with sepsis or poorer neurological outcome
	Docimo et al. (2015)	57	All Trauma (age ≥ 18) undergoing abdominal surgery	(L4/5) VSR, SFA, VFA, PNF	Complications	Complications: VSR (VSR ≥ 0.4 vs. < 0.4, *p* = 0.290) unadjusted VSR continuous (*p* = 0.340) unadjusted VFA (*p* = 0.110) unadjusted SFA (*p* = 0.100) unadjusted PNF (*p* = 0.270) unadjusted	No associations between any of the adipose metrics and complications
	Parenteau et al. (2013)	228	Frontal Crashes	(L2) VFA, SFA	Serious abdominal injury (MAIS ≥ 3)	Serious abdominal injury (MAIS ≥ 3): VFA OR 1.46, 95% CI 1.15–1.85, p < **0.010** SFA OR 0.66, 95% CI 0.52–0.84, p < **0.010**	Higher visceral fat was independently associated with increased risk of serious abdominal injury, whereas greater subcutaneous fat was independently protective against serious abdominal injury after multivariable adjustment.
	Tee et al. (2021)	592	Vulnerable Road Users Trauma	(L2) VFR & (L4) SFR	Serious abdominal injury (MAIS ≥ 3)	Serious abdominal injury (MAISabd ≥ 3): SFR OR 0.063, 95% CI 0.008–0.509, p = **0.009** VFR OR 1.736, 95% CI 0.065–46.464, *p* = 0.742	Greater subcutaneous fat was independently protective against serious abdominal injury supporting the cushion effect.
	Zhang et al. (2013)	118	Frontal Crashes	(L2) VFA	Serious thoracic injury (MAIS ≥ 3)	Serious thoracic injury (MAISthorax ≥ 3): Visceral fat area β = 0.195, 95% CI − 0.422 to 0.811, *p* = 0.350	Higher visceral fat was not independently associated with thoracic injury severity.
Craniofacial	Hu et al. (2018)	108	Traumatic Brain Injury (age ≥ 55)	(2 cm below Zygomatic Arch) MCSA	Pneumonia (Unadjusted group analysis only)	Group analysis only: Ventilator-associated pneumonia—36.0% vs. 24.1% (p = **0.300**) Discharge to home—0% vs. 13.3% (p = **0.040**)	Sarcopenia was associated with poorer functional discharge outcomes, while no statistically significant increase in specific in-hospital complications was demonstrated.
Bone Mineral Density	Armstrong et al. (2022)	336	Motor Vehicle Collision (age ≥ 50)	(L3) SMI/BMD - lumbar BMD < 145 mg/cm³	Complications, ICU Admission, Rehabilitation on discharge	Osteopenia: Any complication: OR not reported ICU admission: OR not reported Discharge to rehabilitation: OR 0.344, 95% CI 0.142–0.833, p = **0.023** Osteosarcopenia: Any complication: OR 0.989, 95% CI 0.453–2.161, *p* = 0.978 ICU admission: OR 0.389, 95% CI 0.165–0.915, p = **0.031** Ventilator support: OR 0.281, 95% CI 0.079–0.999, p = **0.050** Ventilator duration (if ventilated): OR 4.291, 95% CI 1.047–17.591), p = **0.043** Discharge to rehabilitation: OR 1.194, 95%CI 0.542–2.628, *p* = 0.660	Osteosarcopenia was independently associated with predicting ICU admission in the trauma cohort as well as ventilator duration. Osteopenia alone could independently predict whether a patient will be discharged to a rehabilitation facility.
	Kaplan et al. (2016)	450	All Trauma in ICU (age ≥ 65)	(L3) BMD - Trabecular attenuation < 100HU	Complication, Unfavourable discharge	Complications: no statistically significance differences between groups (*p* = 0.850) Discharge disposition: no statistically significance differences between groups (*p* = 0.200)	CT-defined osteopenia was not associated with in-hospital complications or adverse discharge outcomes.
	Parenteau et al. (2013)	228	Frontal Crashes	(L2) TBD, CBD	Serious abdominal injury (MAIS ≥ 3)	TBD: t = − 4.11, *p* < 0.001 unadjusted CBD: t = − 3.09, *p* = 0.004 unadjusted Model-averaged logistic regression (vehicle + morphomics): Trabecular bone density β = 1.251, 95% CI − 0.092 to 2.594, importance = 1.000. Cortical bone density β = −0.447, 95% CI − 1.947 to 1.052, importance = 0.375.	Lower CT-derived trabecular and cortical vertebral bone density were strongly associated with increased odds of serious abdominal injury (MAIS ≥ 3) in frontal vehicle crashes, with trabecular bone density emerging as one of the most important morphomic predictors in multivariable model-averaged analyses.
	Zhang et al. (2013)	118	Frontal Crashes	(L2) TBD, CBD	Serious thoracic injury (MAIS ≥ 3)	TBD: t = 2.705, *p* = 0.008. CBD: t = 1.771, *p* = 0.079. Model-averaged logistic regression (vehicle + morphomics): Trabecular bone density β = −1.754, 95% CI − 2.882 to − 0.626, importance = 1.000. Cortical bone density β = 1.192, 95% CI − 0.136 to 2.520, importance = 0.925.	Lower CT-derived trabecular vertebral bone density was strongly and consistently associated with increased risk of serious thoracic injury (MAIS ≥ 3), emerging as one of the most important morphomic predictors in model-averaged analyses, while cortical bone measures showed weaker and less consistent associations.

Abbreviations: *AM* analytic morphomics, *BAI* brain atrophy index, *BMD* bone mineral density, *CBD* cortical bone density, *CI* confidence interval, *CSA* cross-sectional area, *GOS* Glasgow Outcome Scale, *HR* hazard ratio, *HU* Hounsfield units, *ICU* intensive care unit, *ISS* Injury Severity Score, *LPA* lean psoas area, *MAIS* Maximum Abbreviated Injury Scale, *MCSA* masseter cross-sectional area, *MV* mechanical ventilation, *NOS* Newcastle–Ottawa Scale, *OR* odds ratio, *PCSA* psoas cross-sectional area, *PD* psoas density, *PMI* psoas muscle index, *PMD* psoas muscle density, *PMRA* psoas muscle radiation attenuation, *PNF* perinephric fat thickness, *RR* risk ratio, *RRT* renal replacement therapy, *SAT* subcutaneous adipose tissue, *SFA* subcutaneous fat area, *SMD* skeletal muscle density, *SMI* skeletal muscle index, *TBD* trabecular bone density, *TMA* total muscle area, *TPV* total psoas volume, *UTI* urinary tract infection, *VAT/VFA* visceral adipose tissue/visceral fat area, *VFR* visceral fat ratio, *VSR* visceral-to-subcutaneous fat ratio

Effect estimates: Effect estimates are reported as adjusted values where multivariable models were available. Where adjusted analyses were not performed or not reported, unadjusted estimates, correlation coefficients, group comparisons, or descriptive analyses are presented and explicitly indicated. Regression coefficients (β) represent the change in the outcome per unit change in the predictor unless otherwise specified. Effect estimates are reported with 95% confidence intervals (CI) and corresponding *p* values where available

Not all studies demonstrated complication associations. In older patients with hip fractures, Chang et al. found no relationship between paraspinal muscle density and medical complications or readmission, although lower density was associated with increased transfusion requirements (β = − 0.055 95% CI − 0.093 to − 0.018, *p* = 0.004), suggesting an effect on physiologic reserve rather than discrete complications [28].

## Adiposity analytic morphomics

Seven studies evaluated CT-derived adiposity-related analytic morphomic parameters across heterogeneous trauma and injury populations, including general trauma cohorts, ICU-only populations, elderly hip fracture patients, and motor vehicle collision datasets. Adiposity measures were predominantly derived from single axial CT slices at the thoracolumbar level and focused on visceral fat area or index, subcutaneous fat measures, and visceral-to-subcutaneous ratios. Outcomes included mortality, length of stay (LOS), complications, and injury severity, allowing for outcome-specific synthesis of the prognostic relevance of adipose tissue distribution through AM. Key study characteristics and outcome associations relating to adiposity AM are summarised in Table 6.

**Table 6:** Adiposity analytic morphomics

Title	Author	Date	Cohort (*n*)	Population	Level Measured	Software	Analytic Morphomic Variables	Conclusions
*The association of radiologic body composition parameters with clinical outcomes in level‑1 trauma patients*	Sweet et al.	2023	404	All Trauma	L3	Quantib Body Composition, version 0.2.1 (Quantib, Rotterdam, The Netherlands) – automated segmentation	Visceral fat area (VFA)	Higher visceral fat was independently associated with nearly double the risk of delirium, while showing weaker or non-significant associations with other complications.
*Based on CT scans at the 12th thoracic spine level*,* assessing the impact of skeletal muscle and adipose tissue index on one-year postoperative mortality in elderly hip fracture patients: a propensity score-matched multicentre retrospective study*	Li et al.	2025	334	Hip fracture post-surgery	T12	ImageJ (National Institute of Health Image program v1.52c – manual/semi-automated segmentation)	Visceral fat index (VFI), subcutaneous fat index (SFI), visceral-to-subcutaneous ratio (VSR)	Skeletal muscle index (SMI) measured at T12 was an independent predictor of one-year mortality, while adiposity indices (visceral fat index, subcutaneous fat index, visceral-to-subcutaneous ratio) were not significantly associated with outcomes.
*Impact of Pathologic Body Composition in Multiple Trauma Patients*	Poros et al.	2021	297	Trauma ICU	L3	OsiriX (OsiriX Lite, Pixmeo, Geneva, Switzerland) – semi-automated segmentation	Visceral fat area (VFA), subcutaneous fat area (SFA)	Higher abdominal fat influenced pre-hospital and ED treatment factors (e.g., lower intubation rates, reduced fluid resuscitation, higher transfusion rates), but did not significantly affect ICU stay, ventilation duration, sepsis, or neurologic outcome in multiple trauma patients.
*Utilizing quantitative measures of visceral adiposity in evaluating trauma patient outcomes*	Docimo et al.	2015	57	All Trauma > 18 & Abdominal Trauma requiring surgery	L4/5 intervertebral pace	OsiriX DICOM Viewer (Pixmeo SARL, Geneva, Switzerland) – semi-automated/manual segmentation	Visceral fat area (VFA), subcutaneous fat area (SFA), visceral-to-subcutaneous ratio (VSR), perinephric fat (PNF)	Quantitative visceral adiposity measures (VFA, SFA, and VSR ratio) were not significantly associated with length of stay or postoperative complications, although perinephric fat thickness (PNF) correlated with length of stay on unadjusted analysis.
*Can Anatomical Morphomic Variables Help Predict Abdominal Injury Rates in Frontal Vehicle Crashes?*	Parenteau et al.	2014	228	Frontal Crashes	Multiple levels	Mimics, version 14.11 (Materialise, Leuven, Belgium) – semi-automated/manual segmentation	Visceral fat area (VFA), subcutaneous fat area (SFA).	Visceral fat area, trabecular bone density, and spine angulation, significantly improved prediction of serious abdominal injury in frontal vehicle crashes compared with vehicle and demographic data alone.
*Does a “cushion effect” really exist? A morphomic analysis of vulnerable road users with serious blunt abdominal injury*	Tee et al.	2021	592	All Trauma	L2 & L4	Analytic Morphomics platform (University of Michigan, Ann Arbor, MI, USA) – semi-automated/manual segmentation	Subcutaneous fat ratio (SFR), visceral fat ratio (VFR)	In vulnerable road users, greater subcutaneous fat ratio at L4 was independently protective against serious abdominal injury, supporting the existence of a “cushion effect”.
*Prediction of thoracic injury severity in frontal impacts by selected anatomical morphomic variables through model-averaged logistic regression approach*	Zhang et al.	2013	188	Frontal Crashes	L2	Analytic Morphomics platform (University of Michigan, Ann Arbor, MI, USA) – semi-automated/manual segmentation	Visceral fat area (VFA)	Adiposity-related morphomic measures (particularly visceral fat area and overall body area) were significantly associated with higher risk of severe thoracic injury in frontal crashes, underscoring the prognostic value of fat distribution beyond BMI.

### Mortality

Table 2 summarises mortality outcomes reported for adiposity AM. In a multicentre propensity-matched cohort of elderly hip fracture patients, Li et al. reported no independent association between visceral fat index, subcutaneous fat index, or visceral-to-subcutaneous ratio and 1-year mortality after multivariable adjustment [24]. Although borderline inverse associations were observed on conditional logistic regression (e.g. visceral fat index OR 0.976, 95% CI 0.952–1.001; *p* = 0.063), these effects did not persist following adjustment across institutions.

### Hospital and ICU length of stay

Table 3 summarises LOS outcomes reported for adipose AM. Adiposity metrics demonstrated largely null associations with LOS across studies. In a mixed trauma cohort, Sweet et al. found that CT-derived visceral fat was not independently associated with hospital LOS (β = 1.02, 95% CI 0.93 to 1.12; *p* = 0.690), ICU LOS (β = 1.07, 95% CI 0.81 to 1.43; *p* = 0.620), or duration of mechanical ventilation (β = 1.06, 95% CI 0.86 to 1.31; *p* = 0.550) following multivariable adjustment. Similar findings were reported by Poros et al. in a large ICU-based trauma cohort, where neither visceral- nor subcutaneous adipose tissue was independently associated with ICU LOS or ventilatory duration after adjustment for injury severity and physiological parameters [41].

Docimo et al. similarly reported no associations between visceral or subcutaneous fat areas and LOS in a surgical abdominal trauma cohort, with unadjusted analyses demonstrating no differences when stratified by visceral-to-subcutaneous fat ratio. An exception was observed for perinephric fat thickness, which correlated with longer LOS on unadjusted analysis (*p* = 0.031), although no multivariable modelling was performed, limiting inference regarding independence of this association [45]. Overall, adiposity-based morphomic measures did not independently predict hospital or ICU LOS following adjustment.

### Adverse outcomes, complications, and injury severity

From the six studies included in this section, adiposity metrics showed limited associations with in-hospital complications and adverse outcomes, with notable exceptions for specific endpoints. Table 5 summarises complication and adverse outcome measures reported for adipose AM. In adjusted analyses, Sweet et al. found no independent association between visceral fat and overall complications or infectious morbidity; however, visceral fat was independently associated with delirium, with nearly a twofold increased risk per standard deviation increase (OR 1.95, 95% CI 1.12–3.41, *p* = 0.018) [40]. No associations were observed for unfavourable neurologic outcome or discharge status. Similarly, Poros et al. reported no independent associations between adiposity measures and sepsis or adverse neurologic outcomes after multivariable adjustment [41], while Docimo et al. found no associations between global adiposity measures and postoperative complications, although analyses were limited to unadjusted comparisons [45].

In contrast, adiposity demonstrated more consistent associations when injury severity, rather than post-injury complications, was examined. In frontal motor vehicle collisions, Parenteau et al. reported that visceral fat area independently increased the risk of serious abdominal injury (MAIS ≥ 3) (OR 1.46, 95% CI 1.15–1.85, *p* < 0.010), while subcutaneous fat area conferred a protective effect (OR 0.66, 95% CI 0.52–0.84 *p* < 0.010) [46]. Similar protective effects of subcutaneous adiposity were observed by Tee et al. in vulnerable road users, where higher subcutaneous fat ratio independently reduced the risk of serious abdominal injury (OR 0.063, 95% CI 0.008–0.509, *p* = 0.009), while visceral fat measures were not independently associated [33]. By contrast, Zhang et al. found no independent association between visceral fat area and serious thoracic injury (MAIS ≥ 3) after multivariable adjustment, suggesting that adiposity-related morphomics may influence abdominal but not thoracic injury risk, with thoracic injury severity driven predominantly by crash mechanics and non-adipose morphomic factors [47].

## Craniofacial analytic morphomics

Craniofacial analytic morphomics have gained increasing attention with the widespread use of non-contrast CT brain imaging in trauma. Studies included in this review evaluated craniofacial muscle and bone surrogates of frailty, most commonly masseter cross-sectional area (MCSA), alongside cranial bone and brain metrics such as temporal bone thickness and brain atrophy indices. Outcomes included mortality, hospital and ICU utilisation, and adverse clinical outcomes. Key study characteristics and outcome associations relating to craniofacial AM are summarised in Table 7.

**Table 7:** Craniofacial analytic morphomics

Study Name	Author	Date	Cohort (*n*)	Population	Level Measured	Software	Analytic Morphomic Variables	Conclusions
Morphomic measurement of the temporalis muscle and zygomatic bone as novel predictors of hospital-based clinical outcomes in patients with mandible fracture	Lisiecki et al.	2013	16	Mandible Fracture	Plane through external auditory meatus, superior orbital rim and mandibular coronoid process	MATLAB version 13.0 (MathWorks Inc., Natick, MA, USA) – semi-automated segmentation	Temporal muscle thickness (TMT), zygomatic bone thickness (ZBT), temporalis local mean thickness (TLMT).	Decreased temporalis muscle and zygomatic bone thickness were significantly correlated with longer hospital, ICU, and ventilator days in patients with mandibular fractures, reflecting objective markers of frailty and worse clinical outcomes.
*Sarcopenia as a predictor of mortality in elderly blunt trauma patients: Comparing the masseter to the psoas using computed tomography*	Wallace et al.	2017	487	Blunt Trauma (age ≥ 65)	2 cm below Zygomatic arch	Standard PACS software (DR Systems Web Ambassador, version 8.2; Chicago, IL, USA) – manual segmentation	Masseter cross-sectional area (MCSA)	Masseter muscle cross-sectional area (MCSA) measured on head CT was as valid and more readily available than PCSA in predicting 2-year post-discharge mortality, making it a practical marker of sarcopenia and frailty in this population.
*The associations of psoas and masseter muscles with sarcopenia and related adverse outcomes in older trauma patients: a retrospective study*	Varma et al.	2022	204	All Trauma (age ≥ 65)	2 cm below Zygomatic arch	Carestream Picture Archiving and Communication System (PACS; Carestream Health, Rochester, NY, USA) – manual segmentation	Masseter cross-sectional area (MCSA)	In older trauma patients, psoas sarcopenia (PLVI) predicted higher in-hospital and 2-year mortality and reduced likelihood of discharge home, whereas masseter CSA had no prognostic value.
Sarcopenia diagnosed using masseter muscle area predictive of early mortality following severe traumatic brain injury	Hu et al.	2018	108	Traumatic Brain Injury (age ≥ 55)	2 cm below Zygomatic arch	Local Picture Archiving and Communication System) – manual segmentation	Masseter cross-sectional area (MCSA)	In elderly patients with severe TBI, masseter sarcopenia was strongly associated with higher 30-day mortality and worse discharge outcomes.
Association of Brain Atrophy and Masseter Sarcopenia with 1-year Mortality in Older Trauma Patients.	Tanabe et al.	2019	327	All trauma ICU (age ≥ 65)	2 cm below Zygomatic arch	Local Picture Archiving and Communication System) – manual segmentation	Masseter cross-sectional area (MCSA) & Brain Atrophy Index (BAI)	In older trauma patients, masseter sarcopenia and brain atrophy were independently and cumulatively associated with increased 1-year mortality.

### Mortality

From the 6 studies that report mortality as an outcome, reduced craniofacial muscle mass and structural brain changes were consistently associated with increased longer-term mortality, particularly in older trauma populations. Mortality findings for craniofacial AM are presented in Table 2. In elderly blunt trauma patients, Wallace et al. demonstrated that larger masseter cross-sectional area was independently protective against long-term mortality, with adjusted Cox regression showing a significant reduction in 2-year mortality risk (HR 0.76; 95% CI 0.60–0.96; *p* = 0.023) [23]. Kaplan–Meier survival analyses further demonstrated significantly worse outcomes among patients in the lowest masseter area tertile (*p* = 0.048).

Similarly, Hu et al. reported strong associations between masseter-defined sarcopenia and short-term mortality in older adults with severe traumatic brain injury. Sarcopenic patients experienced significantly higher 30-day mortality (80.0% vs. 50.6%; *p* = 0.010), and sarcopenia remained independently associated with mortality on multivariable logistic regression (OR 2.95, 95% CI 1.03–8.49, *p* = 0.045) [48]. When modelled continuously, increasing masseter area was protective in both logistic (OR 0.66, 95% CI 0.46–0.95, *p* = 0.025) and Cox regression analyses (HR 0.78, 95% CI 0.62–0.97; *p* = 0.042).

The strongest mortality signal emerged when craniofacial muscle and neurostructural metrics were considered together. Tanabe et al. demonstrated that both reduced masseter area (per SD decrease; HR 2.0, 95% CI 1.2–3.1, *p* = 0.005) and increased brain atrophy index (per SD increase; HR 2.0, 95% CI 1.1–3.5, *p* = 0.020) were independently associated with 1-year mortality in trauma ICU patients aged ≥ 65 years [49]. Patients with both masseter sarcopenia and brain atrophy exhibited substantially worse unadjusted survival compared with those with neither condition (HR 3.4, 95% CI 1.5–7.6; *p* = 0.002), indicating an additive effect on long-term mortality risk. No consistent associations were observed for 30-day mortality.

Not all craniofacial muscle measures demonstrated independent prognostic value. Varma et al. found that masseter-defined sarcopenia was not independently associated with either in-hospital mortality (OR 1.18, 95% CI 0.56–5.05, *p* = 0.350) or 2-year mortality (HR 1.76, 95% CI 0.94–3.31, *p* = 0.080), despite strong mortality associations observed with lumbar sarcopenia in the same cohort [37].

### Hospital and ICU length of stay

Associations between craniofacial morphomic measures and hospital utilisation were less consistent and were largely derived from unadjusted analyses. Table 3 summarises LOS outcomes reported for craniofacial AM. Lisiecki et al. reported strong correlations between reduced craniofacial bone and muscle thickness and increased resource utilisation in a small cohort of 16 patients with mandibular fractures [50]. Decreased zygomatic bone thickness was strongly associated with longer hospital stay (*r* = − 0.65; *p* < 0.001), ICU stay (*r* = − 0.57; *p* < 0.001), and ventilator days (*r* = − 0.50; *p* = 0.002), while reduced temporalis muscle thickness similarly correlated with prolonged hospitalisation and ventilator use (*p* < 0.050 for all). However, these findings were based exclusively on unadjusted correlation analyses.

In contrast, studies applying craniofacial muscle metrics in general trauma cohorts reported largely null associations with LOS. Wallace et al. found no meaningful association between masseter area and hospital or ICU length of stay [23], and Varma et al. similarly reported that masseter-defined sarcopenia was not associated with hospital LOS in either unadjusted or adjusted analyses (OR 1.07, 95% CI 0.73–1.59, *p* = 0.720) [37]. ICU LOS was not evaluated in that cohort.

Hu et al. observed that sarcopenic patients with severe traumatic brain injury had numerically longer hospital and ICU stays, although these differences did not reach statistical significance. However, sarcopenic patients experienced significantly fewer hospital-free days within 30 days (*p* < 0.001), reflecting increased early mortality rather than prolonged admission among survivors. All LOS outcomes in this study were derived from unadjusted group comparisons [48].

### Complications and adverse outcomes

Evidence linking craniofacial morphomics to complications and adverse outcomes was limited. Table 5 summarises complication and adverse outcome measures reported for craniofacial AM. In the severe traumatic brain injury cohort studied by Hu et al., sarcopenic patients demonstrated higher rates of ventilator-associated pneumonia (*p* = 0.030) and significantly worse discharge disposition (*p* = 0.040); however, these outcomes were reported descriptively only, with no adjusted analyses performed [48]. No other studies identified independent associations between craniofacial morphomic measures and discrete in-hospital complications.

## Bone mineral density analytic morphomics

CT-derived vertebral bone mineral density (BMD) was evaluated as a marker of frailty, injury severity, and adverse outcomes across a small number of trauma-focused studies, primarily in older or severely injured populations. Metrics predominantly assessed trabecular vertebral attenuation at the lumbar or thoracic level, either alone or in combination with muscle-based morphomics. Key study characteristics and outcome associations relating to BMD AM are summarised in Table 8.

**Table 8:** Bone mineral density analytic morphomics

Study Name	Author	Date	Cohort (*n*)	Population	Level Measured	Software	Analytic Morphomic Variables	Conclusion
Effects of muscle quantity and bone mineral density on injury and outcomes in older adult motor vehicle crash occupants	Armstrong et al.	2022	336	Motor Vehicle Collision (age ≥ 50), Abbreviated injury scale	L1-5	Mimics (Materialise, Leuven, Belgium) – semi-automatic segmentation	Bone mineral density (BMD) using vertebral trabecular bone – mean values from phantomless calibration. Osteopenia < 145 mg/cm^3^	Osteopenia (low lumbar BMD) was linked to higher risk of upper extremity injuries and fractures, and was associated with discharge destination (lower likelihood of rehab placement), while osteosarcopenia mainly influenced ICU/ventilation outcomes
Association of Radiologic Indicators of Frailty With 1-Year Mortality in Older Trauma Patients Opportunistic Screening for Sarcopenia and Osteopenia	Kaplan et al.	2016	450	All Trauma in ICU (age ≥ 65)	L3	Slice-O-Matic, version 5.0 (TomoVision, Montreal, QC, Canada) – semi-automatic/manual segmentation	Bone mineral density (BMD) – vertebral trabecular attenuation. Osteopenia – vertebral trabecular attenuation < 100 HU	Osteopenia was independently associated with increased 1-year mortality, similar in strength to sarcopenia, and together they identified high-risk patients for poor long-term outcomes.
Can Anatomical Morphomic Variables Help Predict Abdominal Injury Rates in Frontal Vehicle Crashes?	Parenteau et al.	2013	228	Frontal Crashes	Multiple	Mimics, version 14.11 (Materialise, Leuven, Belgium) – semi-automated/manual segmentation	Trabecular bone density (TBD) - vertebral trabecular attenuation, cortical bone density (CBD) - full-width-at-half-maximum of the cortical HU signal peak	Lower vertebral trabecular and cortical bone density on CT were significant predictors of serious abdominal injury (MAIS 3+) in frontal crashes, with decreasing BMD linked to higher injury risk
Prediction of thoracic injury severity in frontal impacts by selected anatomical morphomic variables through model-averaged logistic regression approach	Zhang et al.	2013	188	Frontal Crashes	Multiple	Analytic Morphomics platform (University of Michigan, Ann Arbor, MI, USA) – semi-automated/manual segmentation	Trabecular bone density (TBD) - vertebral trabecular attenuation, cortical bone density (CBD) - full-width-at-half-maximum of the cortical HU signal peak	Lower trabecular bone density was independently associated with greater risk of serious thoracic injury (MAIS 3+) in frontal impacts, highlighting BMD as a key predictor of injury severity alongside other morphomic and demographic factors.

### Mortality

Evidence linking CT-derived BMD to mortality was limited but demonstrated strong effects in selected cohorts. In older trauma patients admitted to the ICU, Kaplan et al. reported that osteopenia—defined by low L3 vertebral trabecular attenuation—was independently associated with increased 1-year mortality after adjustment for age, comorbidities, and injury characteristics (HR 11.9, 95% CI 1.3–107.4, *p* = 0.030) [26]. Patients with combined sarcopenia and osteopenia similarly demonstrated significantly increased adjusted mortality risk (HR 9.4, 95% CI 1.2–75.4, *p* = 0.030), supporting vertebral trabecular BMD as an independent prognostic marker of long-term mortality in older trauma populations. Mortality outcomes associated with opportunistic bone mineral density measures are summarised in Table 2.

### Hospital and ICU length of stay

Associations between BMD and length of stay were limited. Table 3 summarises LOS outcomes reported for BMD AM. Kaplan et al. reported no significant differences in hospital or ICU length of stay across CT-defined osteopenia, sarcopenia, combined frailty, or non-frail groups [26]. Length-of-stay outcomes were analysed using univariate group comparisons only, with no adjusted regression modelling performed. Armstrong et al. similarly showed that osteosarcopenia, measured cumulatively (SMI + BMD) at L3 was not independently associated with increased hospital (log transformed β = −0.172, 95% CI − 0.418 to 0.074, *p* = 0.169) or ICU LOS (OR 0.788, 95% CI 0.393–1.580, *p* = 0.503) [51].

### Complications, adverse outcomes, and injury severity

Findings relating BMD to complications and adverse outcomes were mixed and outcome dependent. Table 5 summarises complication and adverse outcome measures reported for BMD AM. In severely injured motor vehicle crash occupants aged ≥ 50 years, Armstrong et al. reported that osteosarcopenia was not associated with overall in-hospital complications after adjustment (OR 0.989, 95% CI 0.453–2.161, *p* = 0.978). However, osteosarcopenic patients were less likely to require ICU admission (OR 0.389, 95% CI 0.165–0.915, *p* = 0.031) or ventilatory support (OR 0.281, 95% CI 0.079–0.999, *p* = 0.050), but those requiring ventilation experienced longer durations of mechanical ventilation (OR 4.291, 95% CI 1.047–17.591, *p* = 0.043). Osteopenia alone was independently associated with a reduced likelihood of discharge to rehabilitation (OR 0.344, 95% CI 0.142–0.833, *p* = 0.023) [51]. Kaplan et al. reported no significant associations between osteopenia and in-hospital complications or discharge disposition, although analyses were descriptive only [26].

BMD demonstrated more consistent associations with injury severity in crash biomechanics studies. Parenteau et al. reported that lower trabecular vertebral bone density was strongly associated with serious abdominal injury (MAIS ≥ 3), with high importance in model-averaged multivariable analyses [46]. Similarly, Zhang et al. demonstrated that reduced trabecular bone density remained a robust predictor of serious thoracic injury (MAIS ≥ 3) (β = −1.754, 95% CI − 2.882 to − 0.626; importance = 1.000), while cortical bone measures showed weaker and less consistent associations [47].

## Discussion

This systematic review demonstrates that CT-derived AM metrics provide valuable prognostic information in trauma populations, extending beyond conventional measures such as chronological age and injury severity scores. Across the included studies, multiple AM domains, including lumbar muscle quantity and quality, adiposity, craniofacial measurements, and opportunistic bone mineral density (BMD), were consistently associated with adverse outcomes such as mortality, length of stay, complications, and functional recovery. However, the degree of predictive value varied depending on the metric employed, the population studied, and the outcomes assessed (Table 5).

Muscle quantity metrics, especially relating to the lumbar muscles, was the most widely investigated AM measure. The majority of studies confirmed that reduced muscle area was significantly associated with increased short- and long-term mortality, especially noted in older and critically ill cohorts. Indices adjusting for height, such as SMI and PMI, offered improved predictive accuracy compared with absolute muscle area, aligning with the recognition that normalising for patient size accounts for interindividual variation. Nevertheless, the predictive value of SMI and PMI was inconsistent across studies; while some reported strong associations with mortality and ICU outcomes, others found only limited or non-significant effects. The introduction of dynamic metrics, such as acute post-traumatic muscle atrophy (ΔPMI), suggests that serial assessment may capture catabolic changes more precisely than static measures [38]. Collectively, these findings support lumbar muscle quantity as a clinically relevant marker of vulnerability, with prognostic performance influenced by population characteristics, measurement approach, and whether longitudinal change was assessed.

Quality metrics, derived from CT attenuation in Hounsfield Units, emphasise tissue integrity and density rather than size. These measures showed a particularly strong and consistent relationship with outcomes. Reduced psoas density was robustly linked to prolonged hospitalisation, complications (notably pneumonia and delirium), and unfavourable neurological outcomes. Of note, two studies demonstrated that muscle density outperformed area-based measures in predicting prognosis, underscoring the importance of tissue quality as a surrogate for physiological reserve [30, 40]. Exceptions were noted in highly specific populations (e.g., older burn patients), suggesting that injury type may influence predictive strength [27].

Adipose AM yielded more heterogeneous findings, demonstrating heterogeneous and outcome-specific prognostic relevance across trauma populations. Adiposity measures showed little association with mortality or length of stay following multivariable adjustment, suggesting limited value for predicting post-injury recovery or resource utilisation. In contrast, visceral fat consistently emerged as a strong predictor of injury severity, particularly for serious abdominal injury in mechanistic motor vehicle collision studies, supporting a role in injury transmission rather than physiological reserve [46, 47]. Subcutaneous adiposity frequently demonstrated neutral or protective associations, reinforcing the proposed “cushion effect” in abdominal trauma. Associations with complications were largely null, with the notable exception of delirium, where increased visceral fat conferred higher risk [33]. Overall, adipose morphomics appear more informative for injury pattern prediction than for downstream clinical outcomes.

Craniofacial AM demonstrated that head CT–derived masseter CSA and potentially temporalis thickness are practical and prognostically relevant surrogates for abdominal muscle measures. As MCSA correlated moderately with psoas metrics, this suggests that craniofacial AM may be particularly valuable in settings where abdominal imaging is not available or required. Studies consistently showed that reduced masseter size was associated with higher short- and long-term mortality, worse functional outcomes, and increased discharge to facilities [23]. Furthermore, studies incorporating brain atrophy (BAI) suggested that combining sarcopenia and neurodegenerative markers may improve risk stratification [49].

CT-derived bone mineral density AM demonstrated limited but potentially important prognostic value in trauma populations, particularly among older and critically injured patients. Evidence linking reduced trabecular BMD to mortality was confined to small ICU-based cohorts but showed strong associations with longer-term mortality, especially when combined with sarcopenia, suggesting an additive frailty effect. In contrast, BMD showed little independent association with hospital or ICU length of stay, and relationships with in-hospital complications were inconsistent and outcome specific. However, vertebral trabecular bone density demonstrated more robust and reproducible associations with injury severity in mechanistic crash studies, predicting serious abdominal and thoracic injuries. Overall, BMD-based morphomics appear more informative for injury susceptibility and long-term vulnerability than for short-term hospital utilisation.

The consistent associations observed across diverse trauma populations highlight the potential for AM to complement injury severity scoring, frailty indices, and conventional clinical parameters in prognostic models. However, as current AM tools remain investigational, their clinical integration will require prospective, multicentre validation studies using standardised analytic methods, with AI-assisted approaches potentially improving consistency and scalability, before widespread adoption can be recommended.

The findings of this review suggest a potential role for analytical morphometric markers in predicting trauma outcomes. These methods require no additional imaging beyond standard trauma protocols. Incorporating morphometric assessment into routine practice could improve prognostication and enable early screening for sarcopenia and osteopenia, particularly in older adults (≥ 65 years). Such risk stratification during admission may enhance clinical planning, reduce morbidity and mortality, and promote cost-effective care. With the growing integration of AI-driven analysis, future trauma imaging protocols may routinely include measures such as SMI or psoas density to provide an automated alert for clinicians regarding future risks and outcomes.

This review has several limitations, foremost among them being study heterogeneity. Differences existed across population cohorts (mechanism, gender, age), imaging protocols, software platforms, definitions of AM variables (PCSA, PMI, PMRA), and different outcomes measured. Due to this level of heterogeneity, a formal systematic review with meta-analysis was deferred. However, despite the inability to conduct a meta-analysis, the review adhered to PRISMA guidelines throughout. Most included studies were retrospective, single-centre studies, introducing potential selection bias and limiting generalisability. Adjustment for confounding factors was inconsistently reported. Furthermore, as most studies originated from high-income settings with established trauma imaging infrastructure, findings may not be transferable to lower-resource environments with limited CT access. Given the rapid evolution of AM over the past decade, some studies relied on manual segmentation, introducing inter- and intra-observer variability. Although AI-based segmentation is emerging, its reliability has not yet been fully validated. While frailty encompasses reduced physiologic reserve and increased vulnerability to stressors across multiple domains, sarcopenia represents the physical dimension of this syndrome, and its presence can therefore be viewed as both a symptom of frailty and a potential mechanistic driver of adverse outcomes.

Taken together, the findings from this review emphasise the potential for integrated opportunistic AM assessment in trauma. By capturing muscle, bone, and adiposity metrics from routine CT imaging, clinicians can derive meaningful prognostic information that may inform triage, anticipate complications, and guide rehabilitation and secondary prevention strategies.

## Conclusion

CT-based AM represent a valuable surrogate for physiological reserve, complementing traditional injury severity scores and clinical frailty measures in predicting outcomes. Multiple AM domains, including lumbar muscle quantity and quality, adiposity, craniofacial measurements, and opportunistic bone mineral density were consistently associated with adverse outcomes including mortality, length of ICU and hospital stay, complications, and functional recovery. Future research should prioritise consensus on standardised AM definitions of sarcopenia to enable robust prognostication and comparability across studies.

## Supplementary Information

Below is the link to the electronic supplementary material.

Supplementary Material 1

## Data Availability

No new original data were generated or analysed in this study. All data that support the findings of this review are derived from previously published studies and are cited in the manuscript.

## Abbreviations

*AI*: Artificial intelligence
*AIS*: Abbreviated Injury Scale
*AM*: Analytic morphomics
*APTMA*: Acute post-traumatic muscle atrophy
*BAI*: brain atrophy index
*BMD*: bone mineral density
*BMI*: body mass index
*CAAST*: CT Abbreviated Assessment of Sarcopenia following Trauma
*CBD*: Cortical bone density
*CT*: computed tomography
*DMG*: Dorsal muscle group
*GOS*: Glasgow outcome score
*HU*: Hounsfield unit
*ICU*: intensive care unit
*ISS*: injury severity score
*LDM*: low density muscle
*LOS*: length of stay
*LPA*: lean psoas area
*MAIS*: Maximum Abbreviated Injury Scale
*MCSA*: masseter cross-sectional area
*PA*: psoas area
*PCSA*: psoas cross-sectional area
*PD*: psoas density
*PLVI*: psoas-lumbar vertebral index
*PMA*: psoas muscle area
*PMD*: Psoas Muscle Density
*PMI*: psoas muscle index
*ΔPMI*: change in psoas muscle index
*PMRA*: Psoas Muscle Radiation Attenuation
*PNF*: perinephric fat thickness
*PRISMA*: Preferred Reporting Items for Systematic review and Meta-analyses
*PSD*: paraspinal muscle density
*SAD*: sagittal abdominal diameter
*SAT*: subcutaneous adipose tissue
*SFA*: subcutaneous fat area
*SFI*: subcutaneous fat index
SFR: subcutaneous fat ratio
*SMD*: skeletal muscle density
*SMI*: skeletal muscle index
*TBD*: trabecular bone density
*TBI*: traumatic brain injury
*TLMT*: temporalis local mean thickness
*TMT*: temporal muscle thickness
*TPV*: total psoas volume
*VAT*: visceral adipose tissue
*VFA*: visceral fat area
*VFI*: visceral fat index
*VRU*: vulnerable road user
*VSR*: visceral-to-subcutaneous fat ratio
*WBCT*: whole-body computed tomography
*ZBT*: zygomatic bone thickness
