# Supplementary material for: Computed tomography derived analytic morphomics as predictors of clinical outcomes in trauma: a systematic narrative review
Source: Emerg Radiol. 2026 Feb 7;33(2):391–422. doi: 10.1007/s10140-026-02441-x (PMC13079524; doi:10.1007/s10140-026-02441-x)
Supplement: Supplementary file 1 — Supplementary Material 1 [file 10140_2026_2441_MOESM1_ESM.docx]

Supplementary Tables:

*Supplementary Table 1: Newcastle Ottawa Score*

|  | Author | Date | Cohort (n) | Total score out of 9 | Representativeness of Exposed Cohort (1) | Selection of Non-Exposed Cohort (1) | Ascertainment of Exposure (1) | Outcome Not Present at Start (1) | Comparability: Controlled for Confounders (Max 2) | Assessment of Outcome (1) | Follow-up Long Enough (1) | Adequacy of Follow-up (1) |
| --- | --- | --- | --- | --- | --- | --- | --- | --- | --- | --- | --- | --- |
| 1 | Tazerout et al. | 2022 | 114 | 7 | 0 | 1 | 1 | 1 | 2 | 1 | 1 | 0 |
| 2 | Sweet et al. | 2023 | 404 | 8 | 1 | 1 | 1 | 1 | 2 | 1 | 1 | 0 |
| 3 | Li et al. | 2025 | 334 | 8 | 1 | 1 | 1 | 1 | 2 | 1 | 1 | 0 |
| 4 | Meyer et al. | 2024 | 472 | 8 | 1 | 1 | 1 | 1 | 2 | 1 | 1 | 0 |
| 5 | Leeper et al. | 2016 | 445 | 9 | 1 | 1 | 1 | 1 | 2 | 1 | 1 | 1 |
| 6 | Yoo et al. | 2017 | 151 | 6 | 1 | 1 | 1 | 0 | 1 | 1 | 1 | 0 |
| 7 | Romanowski et al. | 2021 | 83 | 6 | 0 | 1 | 1 | 1 | 1 | 1 | 1 | 0 |
| 8 | Touban et al. | 2019 | 558 | 9 | 1 | 1 | 1 | 1 | 2 | 1 | 1 | 1 |
| 9 | Chang et al. | 2018 | 91 | 7 | 1 | 1 | 1 | 0 | 2 | 1 | 1 | 0 |
| 10 | Armstrong et al. | 2022 | 336 | 7 | 1 | 1 | 1 | 0 | 2 | 1 | 1 | 0 |
| 11 | Proksch et al. | 2021 | 76 | 8 | 1 | 1 | 1 | 0 | 1 | 1 | 1 | 0 |
| 12 | Poros et al. | 2021 | 297 | 7 | 1 | 1 | 1 | 0 | 2 | 1 | 1 | 0 |
| 13 | Couch et al. | 2017 | 205 | 6 | 1 | 1 | 1 | 0 | 1 | 1 | 1 | 0 |
| 14 | Lisiecki et al. | 2013 | 16 | 5 | 0 | 0 | 1 | 1 | 0 | 1 | 1 | 1 |
| 15 | Badminton et al. | 2025 | 197 | 7 | 1 | 1 | 1 | 0 | 2 | 1 | 1 | 0 |
| 16 | Byun et al. | 2019 | 494 | 9 | 1 | 1 | 1 | 1 | 2 | 1 | 1 | 1 |
| 17 | Byun et al. | 2024 | 217 | 9 | 1 | 1 | 1 | 1 | 2 | 1 | 1 | 1 |
| 18 | Hovsepian et al. | 2024 | 64 | 6 | 1 | 1 | 1 | 0 | 1 | 1 | 1 | 0 |
| 19 | Fairchild et al. | 2015 | 252 | 7 | 1 | 1 | 1 | 0 | 2 | 1 | 1 | 0 |
| 20 | Wallace et al. | 2017 | 487 | 9 | 1 | 1 | 1 | 1 | 2 | 1 | 1 | 1 |
| 21 | McCusker et al. | 2019 | 325 | 7 | 1 | 1 | 1 | 0 | 2 | 1 | 1 | 0 |
| 22 | Shibahashi et al. | 2017 | 74 | 8 | 1 | 1 | 1 | 1 | 2 | 1 | 1 | 0 |
| 23 | Varma et al. | 2022 | 204 | 7 | 1 | 1 | 1 | 1 | 1 | 1 | 1 | 0 |
| 24 | Nishimura et al. | 2020 | 405 | 7 | 1 | 1 | 1 | 0 | 2 | 1 | 1 | 0 |
| 25 | Tee et al. | 2021 | 939 | 7 | 1 | 1 | 1 | 0 | 2 | 1 | 1 | 0 |
| 26 | Docimo et al. | 2015 | 57 | 5 | 0 | 1 | 1 | 0 | 0 | 1 | 1 | 1 |
| 27 | Kaplan et al. | 2016 | 450 | 9 | 1 | 1 | 1 | 1 | 2 | 1 | 1 | 1 |
| 28 | Parenteau et al. | 2013 | 228 | 6 | 1 | 1 | 1 | 0 | 1 | 1 | 1 | 0 |
| 29 | Tee et al. | 2021 | 592 | 6 | 1 | 1 | 1 | 0 | 1 | 1 | 1 | 0 |
| 30 | Zhang et al. | 2013 | 188 | 6 | 1 | 1 | 1 | 0 | 1 | 1 | 1 | 0 |
| 31 | Hu et al. | 2018 | 108 | 7 | 1 | 1 | 1 | 0 | 1 | 1 | 1 | 1 |
| 32 | Tanabe et al. | 2019 | 327 | 9 | 1 | 1 | 1 | 1 | 2 | 1 | 1 | 1 |
